# MALAT1 modulates alternative splicing by cooperating with the splicing factors PTBP1 and PSF

**DOI:** 10.1126/sciadv.abq7289

**Published:** 2022-12-23

**Authors:** Hui Miao, Fan Wu, Yu Li, Chenyu Qin, Yongyun Zhao, Mingfeng Xie, Hongyuan Dai, Hong Yao, Haoyang Cai, Qianhong Wang, Xu Song, Ling Li

**Affiliations:** ^1^ Center for Functional Genomics and Bioinformatics, Key Laboratory of Bio-Resource and Eco-Environment of Ministry of Education, College of Life Sciences, Sichuan University, Chengdu, Sichuan 610064, China.; ^2^ Department of Platform and Technology, lncTAC Company Limited, Chengdu, Sichuan 610219, China.; ^3^ Center of Growth, Metabolism and Aging, Key Laboratory of Bio-Resource and Eco-Environment of Ministry of Education, College of Life Sciences, Sichuan University, Chengdu, Sichuan 610064, China.; ^4^ The First Accredited Outpatient Department of Western General Hospital, Chengdu, Sichuan 610091, China.

## Abstract

Understanding how long noncoding RNAs (lncRNAs) cooperate with splicing factors (SFs) in alternative splicing (AS) control is fundamental to human biology and disease. We show that metastasis-associated lung adenocarcinoma transcript 1 (MALAT1), a well-documented AS-implicated lncRNA, regulates AS via two SFs, polypyrimidine tract–binding protein 1 (PTBP1) and PTB-associated SF (PSF). MALAT1 stabilizes the interaction between PTBP1 and PSF, thereby forming a functional module that affects a network of AS events. The MALAT1-stabilized PTBP1/PSF interaction occurs in multiple cellular contexts; however, the functional module, relative to MALAT1 only, has more dominant pathological significance in hepatocellular carcinoma. MALAT1 also stabilizes the PSF interaction with several heterogeneous nuclear ribonucleoparticle proteins other than PTBP1, hinting a broad role in AS control. We present a model in which MALAT1 cooperates with distinct SFs for AS regulation and pose that, relative to analyses exclusively performed for lncRNAs, a comprehensive consideration of lncRNAs and their binding partners may provide more information about their biological functions.

## Teaser

The lncRNA MALAT1 collaborates with its binding partners to regulate pre-mRNA processing and promote hepatocellular carcinoma.

## INTRODUCTION

In higher eukaryotes, alternative splicing (AS) is a critical regulatory mechanism of gene expression that increases genetic diversity to control development program and response to the environment (*1*). More than 95% of human multiexon-containing genes are estimated to undergo AS under controlled conditions (*2*, *3*), and defects of AS regulation are closely correlated with a wide range of diseases (*4*, *5*). Processing of nuclear precursor mRNAs (pre-mRNAs) is controlled by selective recognition of distinct cis-acting splicing elements by a set of trans-acting splicing factors (SFs), such as the serine/arginine-rich family of nuclear phosphoproteins (SR proteins) and the heterogeneous nuclear ribonucleoparticle (hnRNP) proteins. In addition to conventional SFs, a growing number of long noncoding RNAs (lncRNAs) have emerged as modulators of AS of specific genes, and they appear to function in AS regulation via diverse mechanisms that include modulating the activity of individual SFs (*6*, *7*). The final splicing patterns are also determined by the combinatorial effects of multiple SFs (*8*–*10*). Thus, elucidating how lncRNAs cooperate with different SFs, as well as their general role in AS regulation, is fundamental to human biology and disease.

The pervasive transcription of mammalian genomes leads to production of tens of thousands of lncRNAs (*11*). We and others have demonstrated that lncRNAs play important roles in normal physiology and in many diseases and that most of them achieve their function by participating in the regulation of practically any step of gene expression (*12*–*15*). The metastasis-associated lung adenocarcinoma transcript 1 (MALAT1) is one of these gene expression–controlling lncRNAs, and its aberrant expression has been implicated in the development and progression of many types of human cancers (*16*). MALAT1 is primarily localized to nuclear speckles, the highly dynamic subnuclear domains responsible for storage, modification, and/or assembly of SFs (*17*). Accordingly, it has been proposed to regulate AS by modulating the activation status of SR proteins and by facilitating SR protein shuttling between speckles and the sites of transcription, where splicing occurs (*18*, *19*). In addition, MALAT1 may use certain other mechanisms to regulate pre-mRNA splicing or other steps of gene expression because, by the genome-wide systematic analyses, it was revealed to target nascent pre-mRNAs and transcriptionally active gene loci (*20*–*22*).

Here, we report that MALAT1 can stabilize interaction between the SFs polypyrimidine tract–binding protein 1 (PTBP1, also known as hnRNP I) and PTB-associated SF (PSF), thereby forming a functional module that operates in the regulation of pre-mRNA AS, and show that the functional module might be implicated in the pathogenesis of hepatocellular carcinoma (HCC). Moreover, several hnRNP proteins other than PTBP1, such as hnRNP A1, hnRNP F, and hnRNP U, are found to also interact with PSF with the assistance of MALAT1, strongly suggesting that MALAT1 may has a broad role in AS regulation by orchestrating actions of different hnRNP proteins.

## RESULTS

### MALAT1 is required for maintenance of the PTBP1/PSF interaction

The RNA binding protein PTBP1 is a well-characterized SF (*23*), which appears to be always copurified with PSF (*24*), a multifunctional DNA and RNA binding protein involved in the regulation of numerous cellular mechanisms such as DNA damage response, transcriptional activation, as well as posttranscriptional splicing, stabilization, and export (*25*). By an analysis of cross-linking immunoprecipitation followed by deep sequencing (CLIP-seq), MALAT1 was identified as a direct RNA target for PTBP1 (*26*); our related work also demonstrated an interaction between MALAT1 and PSF (*12*). Thus, these findings together hint a molecular link among MALAT1, PTBP1, and PSF.

**Fig. 1.**
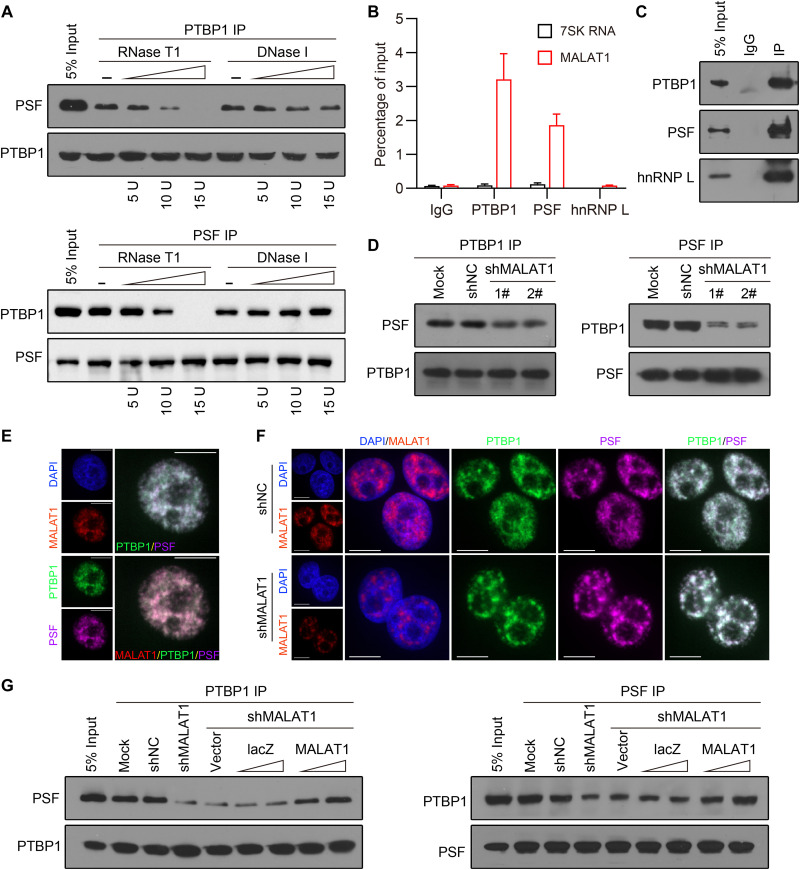
MALAT1 is required for maintenance of the PTBP1/PSF interaction. (**A**) Co-IP assay with antibody (Ab) against PTBP1 (top) or PSF (bottom) detecting the PTBP1/PSF interaction within whole-cell extracts of HEK293 cells and the PTBP1/PSF interaction after RNase T1 or DNase I treatment. The dose of RNase T1 or DNase I was increased as indicated. (**B**) Native RNA IP (RIP) assay followed by reverse transcription quantitative polymerase chain reaction (RT-qPCR) detecting MALAT1 (red bars) and 7SK RNA (black bars) retrieved by PTBP1-, PSF-, or hnRNP L–specific Ab or by normal immunoglobulin G (IgG) in HEK293 cells. Data are shown as means ± standard deviation (SD) of *n* = 3 independent experiments. (**C**) Immunoblotting using the indicated Abs detecting IP efficiency of the native RIP assay in (B). (**D**) Co-IP assay with Ab against PTBP1 (left) or PSF (right) detecting the PTBP1/PSF interaction in HEK293 cells and the PTBP1/PSF interaction after *MALAT1* knockdown by two different short hairpin RNAs (shRNAs). shNC, a nontargeting control shRNA; shMALAT1, a shRNA targeting MALAT1. (**E** and **F**) Confocal images of MALAT1 labeled by RNAscope in situ hybridization (ISH) and PTBP1 and PSF proteins concurrently stained by immunofluorescence (IF) in HEK293 cells (E) and in control and MALAT1-depleted HEK293 cells (F). Scale bars, 10 μm. (**G**) Co-IP assay with Ab against PTBP1 (left) or PSF (right) in HEK293 cells detecting the PTBP1/PSF interaction in response to *MALAT1* knockdown with or without complementation with lacZ or MALAT1 RNA.

We first established a series of co-immunoprecipitation (co-IP) assays to detect and characterize the interaction between PTBP1 and PSF in human embryonic kidney (HEK) 293 cells. The results showed that PTBP1 IP retrieved PSF and, conversely, PSF IP retrieved PTBP1 (Fig. 1A), consistent with the previously reported PTBP1/PSF interaction (*24*). Moreover, treatment with an ascending ribonuclease (RNase) T1 series, rather than a similar treatment with deoxyribonuclease I (DNase I), was revealed to abolish the PTBP1/PSF interaction in cell lysates (Fig. 1A), suggesting that PTBP1 and PSF interact with each other in an RNA-dependent manner.

The antibodies (Abs) against PTBP1 and PSF were used for native RNA IP (RIP) assays. As expected, both of the Abs retrieved MALAT1 transcripts (Fig. 1, B and C). An Ab against another hnRNP protein, hnRNP L, did not retrieved MALAT1, and neither of the above three Abs retrieved 7SK RNA, a nuclear lncRNA implicated in transcription initiation that served as a negative control here (Fig. 1, B and C). We next carried out co-IP assays using HEK293 cells with or without *MALAT1* knockdown. *MALAT1* knockdown was mediated by two independent short hairpin RNAs (shRNAs), which were shown to reduce the level of total MALAT1, act efficiently on the chromatin-associated MALAT1, and have no effect on PTBP1 and PSF levels (fig. S1). Concomitantly, we observed that *MALAT1* knockdown resulted in a substantial decrease, while not a complete abrogation, in the PTBP1/PSF interaction (Fig. 1D). Consistent with above results, colocalization of MALAT1, PTBP1, and PSF was revealed in the nucleus of HEK293 cells by RNAscope in situ hybridization (ISH) and immunofluorescence (IF) costaining (Fig. 1E). Furthermore, we observed marked changes in PTBP1 and PSF subnuclear localization that was caused by *MALAT1* knockdown. In general, PTBP1 and PSF were diffusely distributed throughout the cell nuclei; after MALAT1 depletion, however, the two proteins exhibited a speckle-like distribution pattern (Fig. 1F). Thus, MALAT1 appears to be implicated in maintenance of the PTBP1/PSF interaction.

To further strengthen the above findings, we carried out a co-IP–based compensation experiment by transfecting the *MALAT1*-knockdown HEK293 cells, which exhibited a weakened PTBP1/PSF interaction as expected, with an increasing amount of plasmid expressing lacZ or MALAT1 RNA, with the latter containing several point mutations that disrupt the shRNA target. The exogenous MALAT1, but not the lacZ RNA, was found to have the ability to strengthen the PTBP1/PSF interaction weakened by *MALAT1* knockdown (Fig. 1G). Our results collectively showed that MALAT1 brings PTBP1 and PSF into proximity with each other, thereby forming a unique complex.

### MALAT1 regulates pre-mRNA AS via the bound PTBP1 and PSF

**Fig. 2.**
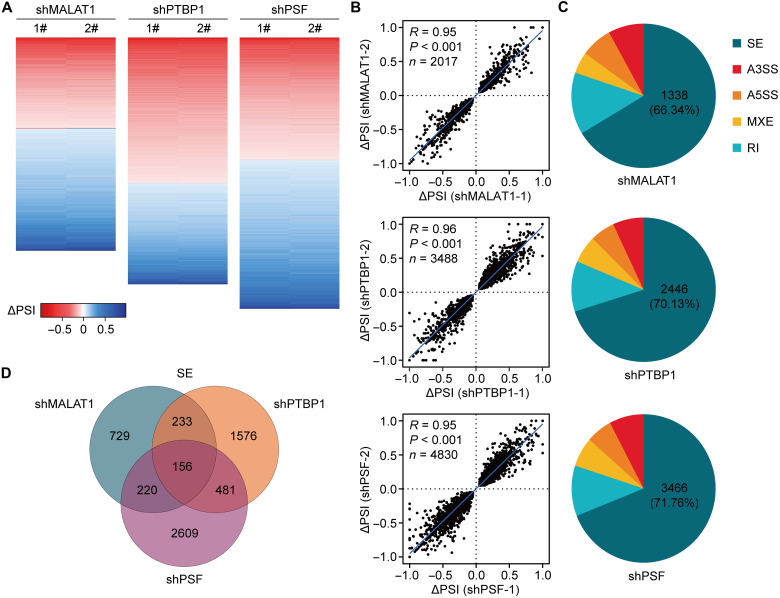
MALAT1 regulates pre-mRNA AS via the bound PTBP1 and PSF. (**A**) Heatmaps showing the changes in percent spliced-in (ΔPSI) compared with the negative control after knockdown of *MALAT1*, *PTBP1*, and *PSF* in HEK293 cells with two independent shRNAs. AS events with significant changes in PSI (*P* < 0.05) are presented. (**B**) Correlation plots showing ΔPSI from two independent MALAT1-, PTBP1-, or PSF-specific shRNAs in HEK293 cells. (**C**) Pie charts showing types of AS events regulated by MALAT1, PTBP1, or PSF depletion as detected by RNA-seq in HEK293 cells. The size of each pie chart reflects the number of events detected. (**D**) Venn diagram showing overlap of cassette exons regulated by MALAT1, PTBP1, and PSF in HEK293 cells.

It is now clear that RNA-protein complex is not only a form of existence of lncRNAs in cells but also represent a functional entity through which those regulatory lncRNAs participate in the construction of regulatory networks in organism (*27*). Because both PTPB1 and PSF can act as SFs, the identified MALAT1-stabilized PTBP1/PSF interaction raises a possibility that MALAT1 may participate in regulation of pre-mRNA AS via the bound PTBP1 and PSF. We first knocked down *MALAT1* with two independent shRNAs in HEK293 cells and subjected the RNA samples for RNA deep sequencing (RNA-seq). Focusing on genes that were regulated at the AS level by both shRNAs, we identified 2017 AS events (representing 1412 genes) that were changed significantly upon *MALAT1* knockdown relative to a nontargeting control shRNA (Fig. 2, A and B). To further evaluate potential involvement of PTBP1 and PSF in the MALAT1-mediated AS regulation, RNA-seq analysis was also carried out for HEK293 cells that underwent *PTBP1* or *PSF* knockdown with two independent shRNAs. The results showed that 3488 and 4830 AS events (representing 2160 and 2813 genes, respectively) were altered significantly by *PTBP1* and *PSF* knockdown, respectively (Fig. 2, A and B).

MALAT1, PTBP1, and PSF were found to affect different types of AS events (Fig. 2C). We first analyzed the most represented category, cassette exons. Of the 1338 MALAT1-regulated exons, 389 (∼29%) and 376 (∼28%) were also regulated by PTBP1 and PSF, respectively (Fig. 2D). As for the 2446 PTBP1-regulated exons, 637 (∼26%) of them were also the downstream targets of PSF (Fig. 2D). A total of 156 exons in 130 genes were revealed to undergo a cooperative regulation by MALAT1, PTBP1, and PSF, which account for ∼12% of the 1338 cassette exons regulated by MALAT1 (Fig. 2D). We randomly selected and confirmed some of the cassette exons using reverse transcription polymerase chain reaction (RT-PCR; fig. S2A). The coordinated AS regulation by MALAT1, PTBP1, and PSF was also observed for other categories of AS events (fig. S2B).

### MALAT1 stabilizes PTBP1/PSF interaction in multiple cellular contexts

**Fig. 3.**
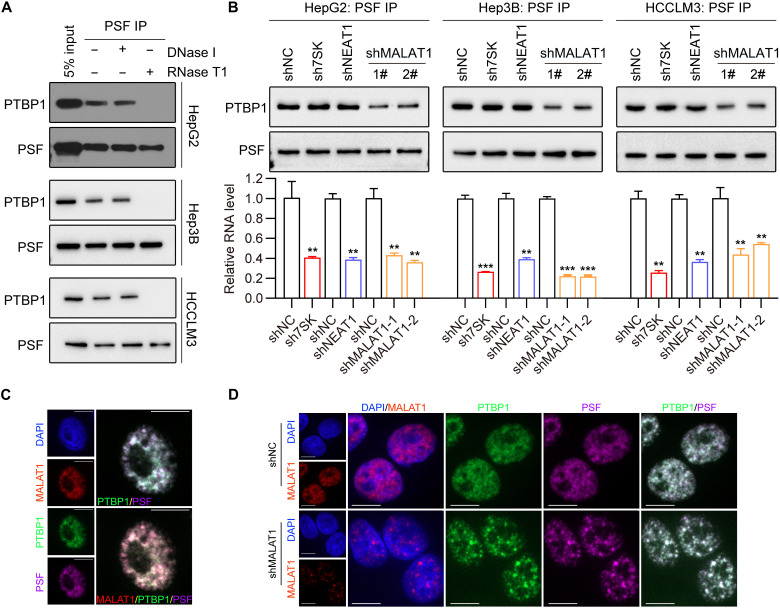
MALAT1 stabilizes PTBP1/PSF interaction in multiple cellular contexts. (**A**) Co-IP assay with anti-PSF Ab detecting the PTBP1/PSF interaction within cell extracts of the indicated HCC cell lines and the PTBP1/PSF interaction after DNase I or RNase T1 treatment. The dose of DNase I and RNase T1 was 15 U per reaction. (**B**) Co-IP assay with anti-PSF Ab detecting the PTBP1/PSF interaction in the indicated HCC cell lines and the PTBP1/PSF interaction after *MALAT1* knockdown by two different shRNAs. Depletion of 7SK RNA and nuclear paraspeckle assembly transcript 1 (NEAT1), two other nuclear-retained lncRNAs used as controls, was found to have no effect on the PTBP1/PSF interaction (top). The knockdown efficiency of 7SK RNA, NEAT1, and MALAT1 was detected by RT-qPCR (bottom). Data are shown as means ± SD of *n* = 3 independent experiments. **P < 0.01 and ***P < 0.001 by Student’s *t* test. (**C** and **D**) Confocal images of MALAT1 labeled by RNAscope ISH and PTBP1 and PSF proteins concurrently stained by IF in HCCLM3 cells (C) and in control and MALAT1-depleted HCCLM3 cells (D). Scale bars, 10 μm.

Following the above studies, we attempted to expand our investigation to other types of cells, especially tumor lines, to seek whether the MALAT1-stabilized PTBP1/PSF interaction, which had been identified in HEK293 cells at the beginning of this research, is a general biological event that can occur in different cellular contexts. By co-IP–based intermolecular interaction analyses, the RNA-dependent and the MALAT1-stabilized PTBP1/PSF interaction was detected in all the tumor cells that we tested (Fig. 3, A and B, and fig. S3). As with the results observed in HEK293 cells, RNAscope ISH and IF costaining also verified the colocalization of MALAT1, PTBP1, and PSF and the changed distribution of PTBP1 and PSF by *MALAT1* knockdown in the nucleus of HCCLM3 cells (Fig. 3, C and D). Thus, our data strongly suggest that rather than HEK293 specific, the simultaneous MALAT1 binding to PTBP1 and PSF should be happened in, and the MALAT1-stabilized PTBP1/PSF interaction might be general for, multiple cellular contexts.

### MALAT1 mediates PSF interaction with several hnRNP proteins other than PTBP1

**Fig. 4.**
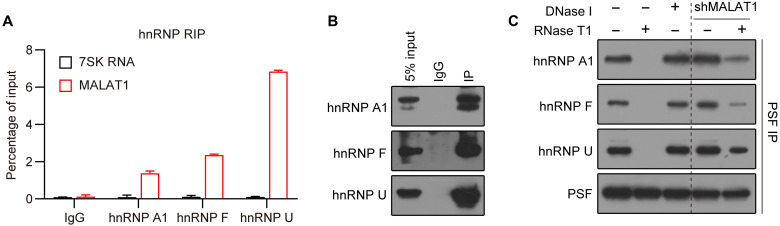
MALAT1 mediates PSF interaction with several hnRNP proteins other than PTBP1. (**A**) Native RIP followed by RT-qPCR detecting MALAT1 and 7SK RNA retrieved by hnRNP A1–, hnRNP F–, or hnRNP U–specific Ab or by normal IgG in HEK293 cells. Data are shown as means ± SD of *n* = 3 independent experiments. (**B**) Immunoblotting using the indicated Abs detecting IP efficiency of the native RIP assay in (A). (**C**) Co-IP assay with anti-PSF Ab detecting the effect of RNase T1 treatment, DNase I treatment, or *MALAT1* knockdown on PSF interaction with the indicated hnRNP proteins in HEK293 cells. The dose of RNase T1 and DNase I was 15 U per reaction.

We subsequently conducted a set of native RIP and co-IP experiments to test several hnRNP proteins other than PTBP1, including hnRNP A1, hnRNP F, and hnRNP U, for their binding to MALAT1 and to PSF in HEK293 cells. The results showed that all the hnRNP proteins can bind to MALAT1 (Fig. 4, A and B) and that the MALAT1 binding can facilitate their interaction with PSF (Fig. 4C). These findings suggest that MALAT1 may act as a platform molecule that provides dispersed recognition sites for the binding of different SFs such as the hnRNP proteins tested here.

### MALAT1, PTBP1, and PSF may act as a functional module in HCC

**Fig. 5.**
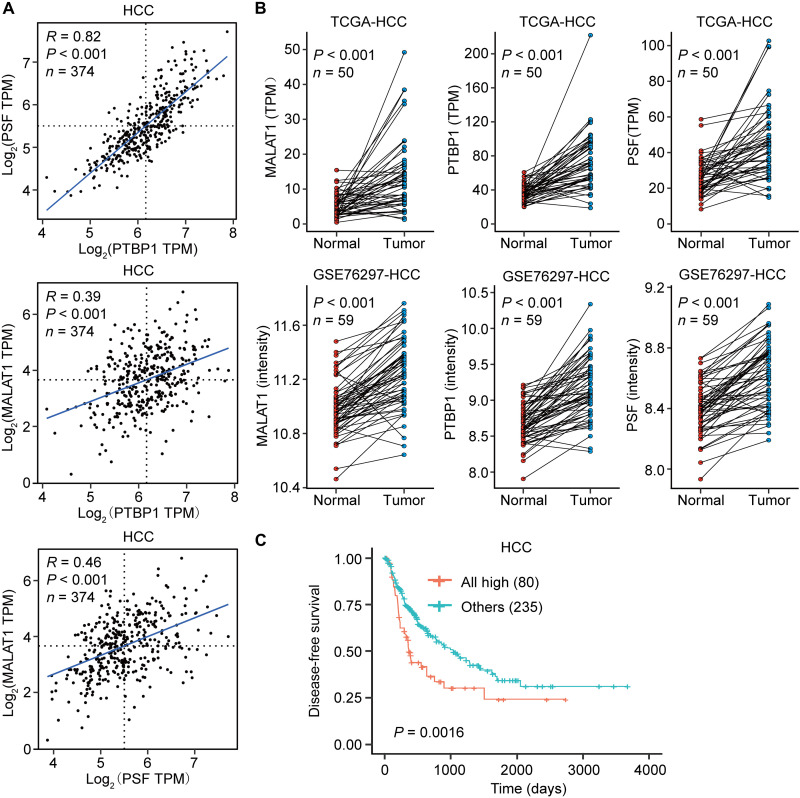
MALAT1, PTBP1, and PSF may act as a functional module in HCC. (**A**) Correlation plots showing expression levels between MALAT1, PTBP1, and PSF in HCC from the TCGA HCC datasets. TPM, transcripts per million. (**B**) Relative expression levels of MALAT1, PTBP1, and PSF in HCC and paired no cancerous hepatic tissue samples from TCGA RNA-seq dataset (top) and GEO GSE76297 dataset (bottom). *P* < 0.001 by Wilcoxon matched-paired signed rank test. (**C**) Kaplan-Meier analysis of the disease-free survival between patients with HCC with high MALAT, PTBP1, and PSF expression levels and others from the TCGA HCC panel. The median expression level was used as the cutoff. The *P* value was determined by log-rank test.

Given that MALAT1, PTBP1, and PSF can interact together to constitute a complex, it seems reasonable to assume that they should be able to act as a functional module if their expression levels maintain a proportional relationship in specific biological contexts. With this in mind, we extracted expression data of MALAT1, PTBP1, and PSF from The Cancer Genome Atlas (TCGA) and analyzed correlation of their expression levels for different types of human cancers, which include HCC, breast invasive carcinoma (BRCA), lung adenocarcinoma (LUAD), colon adenocarcinoma (COAD), and acute myeloid leukemia. We found that expression levels of PTBP1 and PSF are positively correlated with each other in all cancer types (Fig. 5A and fig. S4), thus consistent with the notion that PSF is a PTBP1-associated factor (*24*). Notably, a positive correlation between expression level of MALAT1 and that of PTBP1 and PSF was revealed only for HCC (Fig. 5A and fig. S4). This observation suggests that although formation of a complex appears to be a general biological event for MALAT1, PTBP1, and PSF, the complex might have more dominant biological significance in HCC.

The above findings turned our attention to HCC and prompted an investigation into the expression levels of MALAT1, PTBP1, and PSF in the matched-paired HCC and normal tissue samples in the samples from TCGA RNA-seq dataset and Gene Expression Omnibus (GEO) GSE76297 dataset (Fig. 5B). The results showed that tumor tissue samples exhibit a significant up-regulation of MALAT1, PTBP1, and PSF in these databases, thus suggesting pathological relevance. We further evaluated the prognostic value of *MALAT1*, *PTBP1*, and *PSF* in HCC. While disease-free survival data showed a trend that higher *MALAT1* expression is related to poorer clinical outcome, there was no statistical significance; moreover, a prognostic value was not revealed for either *PTBP1* or *PSF* (fig. S5A). Nevertheless, an interesting finding is that when MALAT1, PTBP1, and PSF are all expressed at a high level, the patients have significantly reduced disease-free survival time compared with others (Fig. 5C). To further corroborate our findings, survival curves were also drawn for BRCA, LUAD, and COAD (fig. S5, B to G), which exhibit no positive correlation between expression level of MALAT1 and that of PTBP1 or PSF (fig. S4). We found that none of *MALAT1*, *PTBP1*, and *PSF* has prognostic value in these cancer types (fig. S5, B to D); in addition, a prognostic value was not observed for their simultaneous high expression (fig. S5, E to G). Thus, our data associate expression levels of MALAT1, PTBP1, and PSF with HCC development and prognosis and strongly suggest that they may act as a functional module in HCC.

### MALAT1 cooperates with PTBP1 and PSF to modulate pre-mRNA AS in HCC cells

**Fig. 6.**
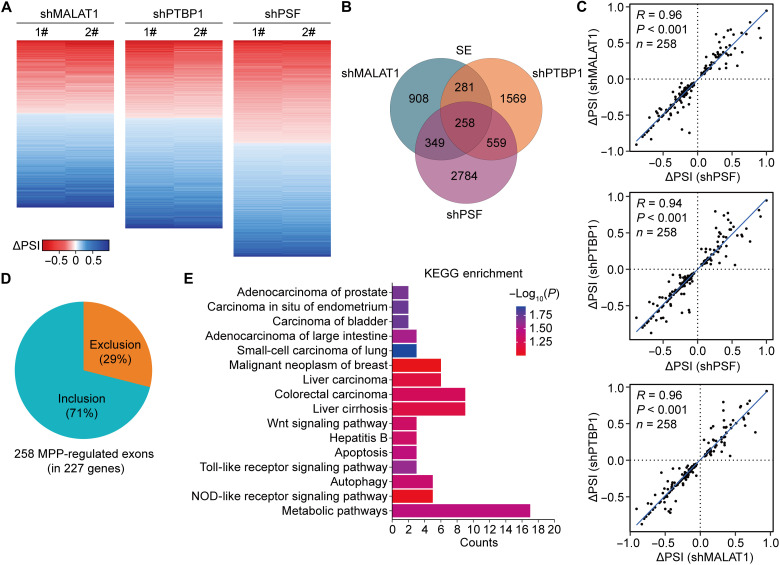
MALAT1 cooperates with PTBP1 and PSF to modulate pre-mRNA AS in HCC cells. (**A**) Heatmaps showing the ΔPSI compared with the negative control after knockdown of *MALAT1*, *PTBP1*, and *PSF* in HepG2 cells with two independent shRNAs. Cassette exons with significant changes in PSI (*P* < 0.05) are presented. (**B**) Venn diagram showing overlap of cassette exons regulated by MALAT1, PTBP1, and PSF in HepG2 cells. (**C**) Correlation plots showing ΔPSI for cassette MPP-regulated exons in HepG2 cells. (**D**) Pie chart showing percentage inclusion and exclusion of the MPP-regulated exons in HepG2 cells. (**E**) Kyoto Encyclopedia of Genes and Genomes (KEGG) pathway analysis of the genes containing MPP-regulated exons. NOD, nonobese diabetic.

Next, we investigated whether MALAT1 also cooperates with PTBP1 and PSF to modulate pre-mRNA AS in HCC cells. We quantified cassette exons by RNA-seq in HepG2 cells and identified 1796, 2667, and 3950 exons with significant changes in splicing after *MALAT1*, *PTBP1*, and *PSF* knockdown, respectively (Fig. 6, A and B). Of the 1796 MALAT1-regulated exons, 258 (∼14%) in 227 genes were found to also undergo a cooperative regulation by PTBP1 and PSF (Fig. 6B and fig. S6A), exhibiting a similar degree of overlap observed in HEK293 cells. The 258 exons coregulated by MALAT1, PTBP1, and PSF (termed MPP-regulated exons hereafter) were positively correlated in their changes in AS (Fig. 6C), with 75 of them (~29%) being induced for exclusion, while the others being induced for inclusion upon MALAT1, PTBP1, and PSF depletion (Fig. 6D). We randomly selected and confirmed some of the MPP-regulated exons using RT-PCR (fig. S6B). Depletion of MALAT1, PTBP1, or PSF was revealed to have no influence on the expression level of the other interaction partners (fig. S6, C to E). Because MALAT1 can be processed to three isoforms (NR_002819.4, NR_144567.1, and NR_144568.1), we designed a set of primers to detect different MALAT1 transcripts using RT–quantitative PCR (RT-qPCR; fig. S6F). We identified all three MALAT1 isoforms in HepG2 cells, with the isoforms 1 and 3 being the two main transcripts (fig. S6G). Nevertheless, levels of the MALAT1 isoforms were not changed by *PTBP1* and *PSF* knockdown (fig. S6H). The findings suggest that these partners do not regulate each other.

A very small portion of MPP-regulated genes overlapped between HepG2 and HEK293 cells (fig. S6I). In HepG2 cells, the MPP-regulated exons were found to be substantially enriched in genes involved in metabolism, liver cirrhosis, hepatitis B, liver carcinoma, and various signaling pathways associated with other cancer types by Kyoto Encyclopedia of Genes and Genomes (KEGG) analysis (Fig. 6E), suggesting a functional implication of the MALAT-stabilized PTBP1/PSF interaction in development and progression of HCC.

### MALAT1 directs association of PTBP1 and PSF with pre-mRNAs

**Fig. 7.**
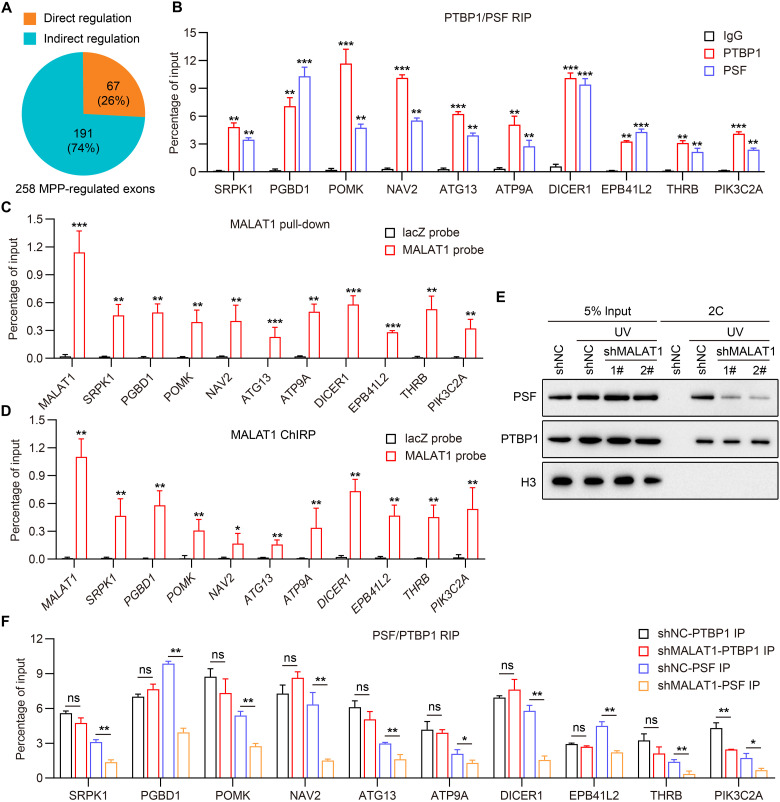
MALAT1 directs association of PTBP1 and PSF with pre-mRNAs. (**A**) Pie chart showing percentage of MPP-regulated exons with or without nearby PTBP1- and PSF-binding sites. (**B**) Native RIP assay followed by RT-qPCR validating the binding of PTBP1 and PSF to the indicated MPP-regulated pre-mRNAs. SRPK1, SRSF protein kinase 1; PGBD1, piggyBac transposable element derived; POMK, protein O-mannose kinase; NAV2, neuron navigator 2; ATG13, autophagy related 13; ATP9A, ATPase phospholipid transporting 9A; DICER1, dicer 1, ribonuclease III; EPB41L2, erythrocyte membrane protein band 4.1 like 2; THRB, thyroid hormone receptor beta; PIK3C2A, phosphatidylinositol-4-phosphate 3-kinase catalytic subunit type 2 alpha. Data are shown as means ± SD of *n* = 3 independent experiments. **P < 0.01 and ***P < 0.001 by Student’s *t* test. (**C**) RNA pull-down followed by RT-qPCR detecting the indicated MPP-regulated pre-mRNAs coprecipitated with MALAT1 in HepG2 cells. Data are shown as means ± SD of *n* = 3 independent experiments. **P < 0.01 and ***P < 0.001 by Student’s *t* test. (**D**) ChIRP assay followed by qPCR detecting the MALAT1 interaction with specific chromatin regions, from which the pre-mRNAs in (C) are produced, in HepG2 cells. Data are shown as means ± SD of *n* = 3 independent experiments. *P < 0.05 and **P < 0.01 by Student’s *t* test. (**E**) 2C analysis detecting the association of PTBP1 and PSF with cellular RNA and the effect of *MALAT1* knockdown on the association. The PTBP1 and PSF association with cellular RNA was dependent on UV cross-link. Histone H3 was used as a negative control that exhibits no association with cellular RNA. (**F**) Native RIP assay followed by RT-qPCR detecting the binding of PTBP1 and PSF to the indicated MPP-regulated pre-mRNAs in control and *MALAT1*-knockdown HepG2 cells. Data are shown as means ± SD of *n* = 3 independent experiments. *P < 0.05 and **P < 0.01 by Student’s *t* test. ns, not significant.

Next, we focused on cassette exons to investigate how MALAT1 participates in AS regulation via the associated PTBP1 and PSF. To this end, we first carried out native RIP followed by deep sequencing (RIP-seq) to identify PTBP1- and PSF-binding sites on pre-mRNAs (fig. S7, A to C, and tables S1 and S2). Intriguingly but expectedly, in silico analyses identified that both PTBP1 and PSF recognize the motifs containing pyrimidine-rich elements (fig. S7D). By an intersection coanalysis of RNA-seq and RIP-seq, 67 (~26%) of the 258 MPP-regulated exons were found to have PTBP1- and PSF-binding sites across their flanking introns and constitutive exons, suggesting direct AS regulation (Fig. 7A and fig. S7E). We referred to pre-mRNAs that have PTBP1- and PSF-binding sites around the MPP-regulated exons as MPP-regulated pre-mRNAs. Ten MPP-regulated pre-mRNAs were selected for native RIP validation of their association with PTBP1 and PSF (Fig. 7B). Moreover, RNA pull-down experiments with biotinylated oligonucleotides antisense to MALAT1 retrieved a substantial amount of MPP-regulated pre-mRNAs that we examined (Fig. 7C), and chromatin isolation by RNA purification (ChIRP) assays revealed that MALAT1 interacted with the corresponding gene loci from which the pre-mRNAs are produced (Fig. 7D). This is coincided with the previous finding that MALAT1 targets both nascent pre-mRNAs and transcriptionally active chromatin (*20*–*22*).

To further define the underlying mechanism, we investigated whether MALAT1 can affect the PTBP1 and PSF association with cellular RNA using a complex-capture method (2C), which involves in cellulo cross-linking of direct RNA-protein interactions by ultraviolet (UV) irradiation, followed by several procedures such as cell lysis, nucleic acid purification on a silica matrix-based column, and immunoblotting assay for specific RNA-bound proteins (*28*). Using 2C on HepG2 cells, we found that *MALAT1* knockdown led to a substantial decrease in the level of RNA-associated PSF (Fig. 7E). In contrast, the association between PTBP1 and cellular RNA was not changed after MALAT1 depletion (Fig. 7E). Moreover, the above MPP-regulated pre-mRNAs were subjected to native RIP assay to strengthen this finding. As shown in Fig. 7F, while MALAT1 could not affect the PTBP1 binding to most of the pre-mRNAs, all pre-mRNA binding by PSF was significantly weakened after MALAT1 depletion. Because PTBP1 can act as a splicing regulator via its direct binding to intronic sequences within pre-mRNAs (*29*), MALAT1 should have a weak ability to affect the PTBP1 binding to pre-mRNA in these cases. Collectively, our data suggest that MALAT1 can interact together with PTBP1 and PSF to form a functional module and that, after the module formation, MALAT1 may direct localization of PSF, as well as that of PTBP1 in some cases, on pre-mRNAs, thereby operating in the modulation of pre-mRNA AS in HCC cells.

### MALAT1, PTBP1, and PSF have synergistic effect on malignant behavior of HCC cells

Following on from the above findings, we set out to analyze the contribution of MALAT1, PTBP1, and PSF to the malignant property of HCC cells. Knockdown of *MALAT1*, *PTBP1*, and *PSF* in HepG2 cells significantly inhibited cell growth, as judged by Cell Counting Kit-8 (CCK-8) assays (fig. S8A). In addition, their depletion markedly impaired the migratory and invasive capacities of cells, as judged by transwell migration/invasion assays (fig. S8, B and C). Moreover, a set of similar knockdown experiments was found to produce similar results in HCCLM3 cells (fig. S8, D to F). To further investigate the tumorigenic function of MALAT1, PTBP1, and PSF in vivo, HepG2 cells stably depleting MALAT1, PTBP1, and PSF or control cells were injected subcutaneously into athymic nude mice. Both the tumor volume and weight showed remarkable reduction in the target depletion groups compared with the control group (fig. S8, G to I), indicating that stable knockdown of *MALAT1*, *PTBP1*, and *PSF* effectively inhibited tumor growth in vivo.

**Fig. 8.**
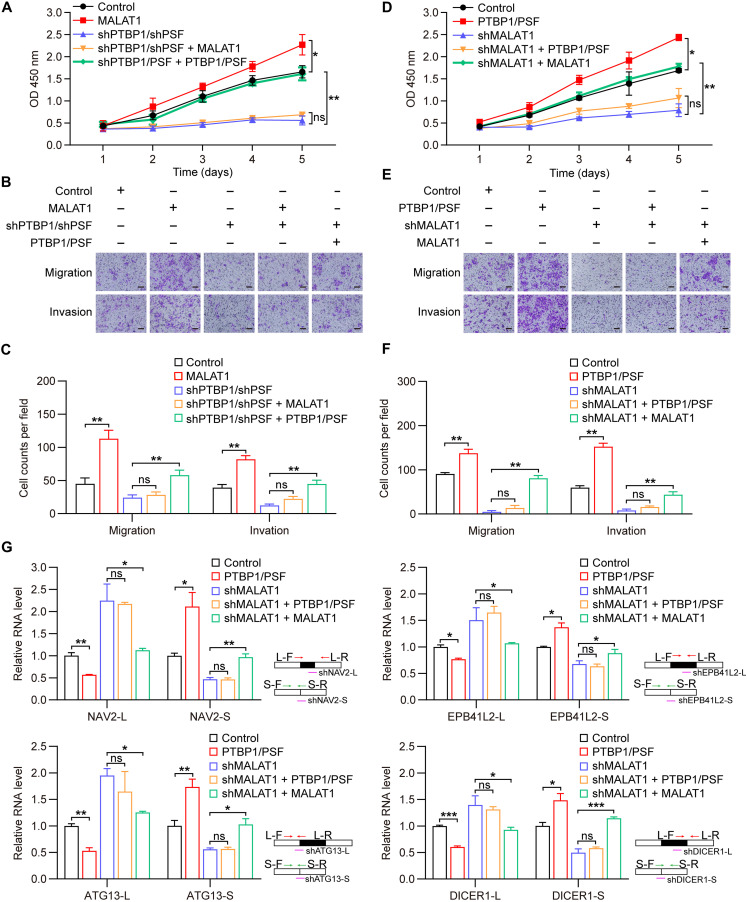
MALAT1, PTBP1, and PSF have synergistic effect on malignant behavior of HCC cells. (**A**) CCK-8 assays detecting implication of PTBP1 and PSF in the regulatory effect of MALAT1 on proliferation of HepG2 cells. Data are shown as means ± SD of *n* = 3 independent experiments. *P < 0.05 and **P < 0.01 by one-way analysis of variance (ANOVA). (**B** and **C**) Transwell assays detecting implication of PTBP1 and PSF in the regulatory effect of MALAT1 on migration [B (top) and C] and invasion [B (bottom) and C] of HepG2 cells. Scale bars, 100 μm. Data in (C) are shown as means ± SD of *n* = 3 independent experiments. **P < 0.01 by one-way ANOVA. (**D**) CCK-8 assays detecting contribution of MALAT1 to the regulatory effect of PTBP1 and PSF on proliferation of HepG2 cells. Data are shown as means ± SD of *n* = 3 independent experiments. *P < 0.05 and **P < 0.01 by one-way ANOVA. (**E** and **F**) Transwell assays detecting contribution of MALAT1 to the regulatory effect of PTBP1 and PSF on migration [E (top) and F] and invasion [E (bottom) and F] of HepG2 cells. Scale bars, 100 μm. Data in (F) are shown as means ± SD of *n* = 3 independent experiments. **P < 0.01 by one-way ANOVA. (**G**) RT-qPCR detecting contribution of MALAT1 to the regulatory effect of PTBP1 and PSF on pre-mRNA AS in HepG2 cells. Primers used for the RT-qPCR detection of long (L) and short (S) transcript variants, as well as shRNAs targeting long and short variants used in fig. S9G, are illustrated. Data are shown as means ± SD of *n* = 3 independent experiments. *P < 0.05, **P < 0.01, and ***P < 0.001 by Student’s *t* test.

Because MALAT1, PTBP1, and PSF had been proposed to act as a functional module in HCC, we attempted to verify this hypothesis by determining whether the regulatory effects of MALAT1 on the malignant biological behavior of HepG2 and HCCLM3 cells are dependent on PTBP1 and PSF. Specially, the procedure involved overexpressing MALAT1 in cells and overexpressing MALAT1 in PTBP1/PSF-depleted cells. MALAT1 overexpression has the ability to make a significant promotion in cell growth, migration, and invasion; the ability, however, was almost completed abrogated by PTBP1/PSF double depletion (Fig. 8, A to C, and fig. S9, A to C). As an experimental control, the effects of PTBP1/PSF depletion were shown to be rescued by PTBP1/PSF restoration (Fig. 8, A to C, and fig. S9, A to C). We also explored whether MALAT1 was a dominant contributor to the regulatory effects of PTBP1 and PSF, and the procedure involved overexpressing PTBP1 and PSF in cells and overexpressing PTBP1 and PSF in MALAT-depleted cells. Similar to the results from the first procedure, PTBP1 and PSF overexpression was revealed to promote cell growth, migration, and invasion, whereas MALAT1 depletion significantly abolished the effects (Fig. 8, D to F, and fig. S9, D to F). As an experimental control, the effect of MALAT1 depletion was shown to be rescued by MALAT1 restoration (Fig. 8, D to F, and fig. S9, D to F).

We selected several splicing events coregulated by MALAT1, PTBP1, and PSF for function investigation but found that none of them can individually contribute to the malignant behavior of HCC cells (fig. S9G). The results might be reasonable because the altered splicing events that we tested may function in a synergistic manner and then act together in HCC cells. In addition, we carried out RT-qPCR using the above MALAT1/PTBP1/PSF-manipulated HepG2 cells for AS analyses. The results showed that PTBP1 and PSF exert regulatory effects on pre-mRNA AS in a MALAT1-dependent manner (Fig. 8G), thereby corroborating our findings obtained from above phenotypic studies at the molecular level. Collectively, our data suggest that MALAT1, PTBP1, and PSF modulate malignant behavior of HCC cells in a synergistic manner, which is coincided with our proposal that MALAT1, PTBP1, and PSF may act as a functional module in HCC.

## DISCUSSION

Understanding how lncRNAs cooperate with different SFs, as well as their general role in AS regulation, is fundamental to human biology and disease. In the present study, we show that MALAT1, one of the first identified AS-implicated lncRNAs, can stabilize interaction between the SFs PTBP1 and PSF, thereby forming a functional module that affects a network of AS events. Moreover, this module may have pathogenic functions in HCC.

PTBP1 and PSF have long been realized to interact with each other (*24*). However, whether and how they coordinately participate in AS regulation still remains an issue to be addressed. While PTBP1 and PSF were ever considered as two partners that may interact with each other directly (*24*, *30*), our current data provide unbiased evidence that the lncRNA MALAT1 is critical and sufficient for maintenance of the PTBP1/PSF interaction. MALAT1 is just one of the PSF-binding lncRNAs as revealed by our related study (*12*), and PTBP1 also interacts with other lncRNAs such as H19 (*31*), suggesting that certain lncRNAs other than MALAT1 may also facilitate the reciprocal interaction between PTBP1 and PSF. In accordance with this notion, current data showed that an RNase T1 treatment, relative to the *MALAT1* knockdown, exhibits more prominent influence on PTBP1/PSF interaction in HEK293 cells and in a series of tumor cells.

MALAT1 is found to be enriched in nuclear speckles, the nonmembranous subnuclear structures that are thought to be the concentrated reservoir of SFs rather than represent major sites of transcription or splicing. In general, posttranscriptional processing of most pre-mRNAs can occur concomitantly with their transcription at transcription sites that preferentially localize within the vicinity of nuclear speckles (*17*). During the two coupled gene expression processes, MALAT1 has been assumed to play a critical role in the shuttling of SR proteins between the speckles and transcription sites (*18*, *19*). Somewhat consistent with this notion, a subsequent study showed that MALAT1 is involved in the organization of transcriptionally active genes at the nuclear speckle periphery (*32*). Moreover, MALAT1 has also been shown to specifically target actively transcribed gene loci and interact with many nascent pre-mRNAs (*20*–*22*). While these observations suggest that MALAT1 can achieve its regulatory functions at the sites of transcription, the underlying molecular mechanism and related biological significance are not yet clear (*33*). From the data described here, we suggest that MALAT1 transcripts, especially those tethered to chromatin, can direct association of the SFs PTBP1 and PSF with pre-mRNAs via a simultaneous interaction with them, thereby operating in the regulation of a network of AS events. Given that many chromatin-tethered lncRNAs have been identified (*27*), more efforts should be needed to investigate their potential implication in pre-mRNA AS regulation and decipher the related biological significance.

Pre-mRNAs are generally recognized by a set of different SFs, and multiple SFs always exhibit combinatorial effects to determine the final splicing outputs (*8*–*10*). Thus, our study provides a possible molecular explanation for the way through which multiple SFs colocalize and then act together on pre-mRNAs. It seems unlikely that PTBP1 is the only hnRNP protein operating in the MALAT1-mediated AS regulation. For instance, several hnRNP proteins other than PTBP1, including hnRNP A1, hnRNP F, and hnRNP U, were found to also interact with MALAT1 and PSF (Fig. 4). These hnRNP proteins presumably act together with the bound MALAT1 and PSF to modulate distinct subsets of AS events. Therefore, we propose that, in addition to its role in modulating the subcellular distribution of SR proteins (*18*, *19*), MALAT1 may have a broad role in AS regulation by orchestrating actions of different SFs, especially those bound by MALAT1, at the sites of transcription.

As reported here, the functional module consisting of MALAT1, PTBP1, and PSF may have pathogenic implications in HCC. While MALAT1 is dysregulated in many types of human cancers and has been proposed as a “broad-spectrum” prognostic marker for poor clinical outcomes, its mechanisms of action in individual cases appear to be different and are dependent on cancer types (*34*). For instance, MALAT1 can function through sponging miRNAs, thus serving as a competitive endogenous RNA, in nasopharyngeal carcinoma and gastric cancer (*35*, *36*); alternatively, it can regulate transcription of a set of metastasis-associated genes in lung cancer (*37*). By analyzing their expression levels, PTBP1 and PSF are revealed to be positively correlated in all cancer types that we have assessed; however, MALAT1 shows a positive correlation with the two SFs only in HCC (Fig. 5A and fig. S4). Moreover, although the expression level of MALAT1, PTBP1, or PSF exhibits nearly no impact on the survival rate of patients with HCC when they are considered independently (fig. S5A), their simultaneous high expression was found to be correlated with a poor patient prognosis (Fig. 5C). We demonstrate that the synergistic effect of MALAT1, PTBP1, and PSF is required for maintenance of the malignant behavior of HCC cells (Fig. 8 and fig. S9). Together, our results not only reinforce the notion of MALAT1 being functionally diverse in human cancers but also indicate that the functional module consisting of MALAT1, PTBP1, and PSF should be implicated in the pathogenesis of HCC. Of course, MALAT1 may also function in HCC through other mechanisms (*38*), and we cannot exclude the possibility that the functional module reported here may play roles in other types of human cancers as well.

In summary, our current study presents a model in which MALAT1 cooperates with the SFs PTBP1 and PSF to form a functional module that directs PTBP1 and PSF association with pre-mRNAs and regulates a network of AS events, providing new insights into mechanisms of MALAT1 in the regulation of gene expression. In addition, MALAT1 was demonstrated to interact with several hnRNP proteins other than PTBP1 and facilitate their interaction with PSF. Although the related biological significance warrants further studies, it could be proposed that MALAT1 may have a broad role in AS regulation by orchestrating actions of different SFs. Moreover, despite the fact that MALAT1 is considered as a broad-spectrum tumor-promoting transcript, the functional module consisting of MALAT1, PTBP1, and PSF appears to have greater clinical value in HCC. Thus, an intriguing idea raised from this study is that, relative to the analysis exclusively carried out for lncRNAs, a comprehensive consideration of both lncRNAs and their binding partners, such as the stoichiometric relationship between them, should be able to provide more detailed and valuable information about their function in human physiological and pathological processes.

## MATERIALS AND METHODS

### Plasmid construction

All plasmids were constructed with restriction-enzyme digestion and ligation method. For lentivirus-mediated RNA interference, complementary sense and antisense oligonucleotides (ASOs) encoding shRNAs targeting MALAT1, PTBP1, and PSF transcripts were synthesized, annealed, and cloned into the Age I and Eco RI sites of plasmid pLKO.1 (Addgene). To obtain the full-length MALAT1, eight head-to-tail overlapping MALAT1 cDNA fragments were synthesized and subjected to a series of overlapping PCR amplifications, and the resultant MALAT1 cDNA was cloned into the Nhe I and Apa I sites of plasmid pcDNA3.1 (Invitrogen). For simultaneous PTBP1/PSF expression, the PTBP1 cDNA was cloned into the Eco RI and Xho I sites of plasmid pLVX–internal ribosomal entry site (IRES)–ZsGreen1 (BD Clontech), and the PSF cDNA was cloned into the Xho I and Age I sites to replace the ZsGreen gene on plasmid. The oligonucleotides encoding shRNAs are shown in table S3.

### Cell culture

HEK293, HepG2, HCCLM3, Hep3B, HuTu-80, HCT116, A549, H1299, T-47D, MCF7, HL60, and THP-1 cells were obtained from the American Type Culture Collection and cultured in Dulbecco’s minimum essential medium, RPMI 1640, or K12-F supplemented with 10% fetal bovine serum (FBS) in a 5% CO_2_ incubator at 37°C. Plasmid transfection was performed using Lipofectamine 2000 (Invitrogen). For lentivirus infection, shRNA-encoding pLKO.1, or pLVX-IRES encoding PTBP1 and PSF, was cotransfected with psPAX2 and pMD2.G plasmids (Addgene) into HEK293 cells; the infectious lentivirus was harvested 2 days after transfection, filtered through 0.45-μm polyvinylidene difluoride (PVDF) filters, and transduced into specific cells. After plasmid transfection or lentivirus infection, the resulting cell population, but not the isolated single clones, was used for subsequent assays to avoid clone-specific effects.

### Antibodies

The Abs used for immunoblotting were rabbit anti-PTBP1 Ab (382421, Zen Bioscience), rabbit anti-PSF Ab (15585-1-ap, Proteintech), rabbit anti-H3 Ab (17168-1-ap, Proteintech), mouse anti–hRNP L Ab (ab6106, Abcam), rabbit anti–hRNP U Ab (ab172608, Abcam), rabbit anti–hRNP A1 Ab (ab208026, Abcam), mouse anti–hRNP F Ab (04-1462, Sigma-Aldrich), and mouse anti-actin Ab (66009-1-Ig, Proteintech). The Abs used for co-IP were mouse anti-PTBP1 Ab (MABE986, Sigma-Aldrich) and mouse anti-PSF Ab (P2860, Sigma-Aldrich). The Abs used for RIP analysis were mouse anti-PTBP1 Ab (MABE986, Sigma-Aldrich), mouse anti-PSF Ab (P2860, Sigma-Aldrich), mouse anti–hRNP L Ab (ab6106, Abcam), rabbit anti–hRNP U Ab (ab172608, Abcam), rabbit anti–hRNP A1 Ab (ab208026, Abcam), mouse anti–hRNP F Ab (04-1462, Sigma-Aldrich), and mouse normal immunoglobulin G (IgG; sc-2025, Santa Cruz Biotechnology). The Abs used for IF were mouse anti-PTBP1 Ab (MABE986, Sigma-Aldrich), rabbit anti-PSF Ab (15585-1-AP, Proteintech), fluorescein isothiocyanate–conjugated donkey anti-mouse IgG (SA00003-9, Proteintech), and CoraLite 647–conjugated donkey anti-rabbit IgG (SA00014-7, Proteintech).

### RT-qPCR detection of gene expression

Total RNA was isolated from cultured cells using TRIzol reagent (Invitrogen). First-strand cDNA was generated using the PrimeScript RT Reagent Kit (TaKaRa). RNA levels for a specific gene were measured by RT-qPCR (starting with 50 to 100 ng of RNA sample per reaction) using Real-Time PCR Easy (Foregene), in accordance with the manufacturer’s instructions. The RT-qPCR data were normalized to beta-actin (ACTB) mRNA. The primers used in the RT-qPCR are shown in table S3.

### Co-IP and immunoblotting

Co-IP and immunoblotting were performed as previously descried with some modifications (*14*). Briefly, a total of 5 × 10^6^ cells were washed twice in cold phosphate-buffered saline (PBS) and pelleted. The pellet was resuspended in lysis buffer [10 mM tris-HCl (pH 7.4), 150 mM NaCl, 0.5% NP-40, 1 mM EDTA, 10 μM dithiothreitol (DTT), and 1 mM phenylmethylsulfonyl fluoride], incubated on ice with frequent vortexing for 15 min, and then the lysate was obtained by centrifugation at 12,000*g* for 10 min. Protein concentrations of the extracts were measured by the bicinchoninic acid assay (Pierce). Four hundred micrograms of the protein was used for IP or was fractionated by SDS–polyacrylamide gel electrophoresis (PAGE), transferred onto PVDF membranes, and then blotted.

For IP assays, protein samples with or without nuclease treatment were incubated with a specific Ab or control IgG overnight at 4°C. Subsequently, the samples were incubated with 50 μl of protein G magnetic beads (Thermo Fisher Scientific) for 1 hour at 4°C and then washed three times in lysis buffer. Last, protein complexes were eluted by SDS buffer [50 mM tris-HCl (pH 6.8), 10% glycerol, 1% β-mercaptoethanol, 0.1% bromophenol blue, and 2% SDS] and then detected by immunoblotting.

### Native RIP assay

Native RIP assay was performed as previously described with some modification (*14*). Briefly, RNase OUT (50 U/ml; Invitrogen) and a protease inhibitor cocktail (PIC; Roche) were added to the lysis buffer (the buffer mentioned here is the same as that used in co-IP and immunoblotting). After an incubation with specific Ab or control IgG overnight at 4°C, samples were incubated with 50 μl of protein G magnetic beads for 1 hour at 4°C and then washed three times in lysis buffer. The beads were resuspended and treated with proteinase K at 45°C for 45 min. Coprecipitated RNAs were extracted using TRIzol reagent (Invitrogen), ethanol-precipitated with GlycoBlue (Invitrogen) as a carrier, and then detected by RT-qPCR. The data of retrieved RNAs are presented as a percentage of the amount input. The primers used for RT-qPCR following native RIP detecting specific RNA transcripts are shown in table S3.

### Chromatin isolation for preparation of chromatin-enriched RNA

A total of 1 × 10^7^ cells were washed twice in ice-cold PBS and then incubated in hypotonic buffer [10 mM tris-HCl (pH 7.5), 10 mM NaCl, 3 mM MgCl_2_, and 0.3% NP-40, supplemented with RNase OUT (50 U/ml) and a PIC] on ice for 10 min. After 5 min of centrifugation at 2000*g*, the supernatant was collected as the cytoplasmic fraction, and after additional washing, the remainder was considered as nuclei pellet. After washing three times with PBS, the nuclei pellet was resuspended and incubated in modified Wuarin-Schibler (MWS) buffer [10 mM tris-HCl (pH 7.0), 4 mM EDTA, 150 mM NaCl, and 0.5% NP-40] on ice for 15 min. The chromatin pellet was collected by 5 min of centrifugation at 1000*g*, and the supernatant was kept as nucleoplasmic fraction. After washing three times with MWS, TRIzol reagent was added to the chromatin pellet to isolate the chromatin-enriched RNA.

### RNAscope ISH and IF costaining

The colocalization of MALAT1, PTBP1, and PSF in HEK293 and HepG2 cells was measured using the RNAscope Multiplex Fluorescent Kit v2 and RNA-Protein Co-Detection Ancillary Kit (Advanced Cell Diagnostics) according to the manufacturer’s instructions. Briefly, cells cultured on coverslips were fixed, permeabilized, and digested by protease to allow target accessibility. After an overnight incubation of Abs against PTBP1 and PSF, a probe set specific for MALAT1 (designed and supplied by Advanced Cell Diagnostics) was added to the cells and hybridization was performed at 40°C for 2 hours. After a series of signal amplification with reagents supplied in the kit, cells were counterstained with 4′,6-diamidino-2-phenylindole (DAPI) and then detected using an inverted confocal microscope (Leica).

### RNA sequencing

Total RNA and RNA purified by native RIP were used for RNA-seq. RNA-seq was performed as previously descried with some modifications (*39*). Briefly, the concentration and integrity of RNA were estimated using the Qubit 2.0 Fluorometer (Invitrogen) and Agilent 2100 Bioanalyzer (Agilent Technologies), respectively. Approximately 1.5 μg of RNA from each sample was used for mRNA library construction. The TruSeq Stranded mRNA Library Prep Kit (Illumina) was used to prepare next-generation sequencing library following the manufacturer’s protocol. Accurate quantification for sequencing applications was determined using the qPCR-based KAPA Biosystems Library Quantification Kit (KAPA Biosystems). Paired-end sequencing [150 base pairs (bp)] was performed on a HiSeq 4000 platform (Illumina) by the Beijing Allwegene Technology Company Limited.

### RNA-seq and RIP-seq data analysis

Raw reads of fastq format were first processed to obtain clean reads, which were aligned to the human genome hg38 using STAR (*40*). The tool ASprofile (https://ccb.jhu.edu/) was used to classify the AS events using the RNA-seq data. The differential AS events were identified by rMARS (*41*), and the output was filtered by significance (*P* < 0.05), false discovery rate (FDR < 0.05), and changes of percent spliced-in (PSI) (|ΔPSI| > 0.05). The online database for annotation, visualization, and integrated discovery containing Gene Ontology and KEGG pathway analysis was used to analyze the biological characteristics and function annotation of candidate genes (*42*). For the RIP-seq analysis, reads uniquely mapping to the genome were used for peak calling with Homer (*43*), assuming the size of 500, extending the peaks to cover the full enriched region, and assuming a fold enrichment of at least 1.5 over input reads and a Poisson *P* value threshold relative to input count of 1 × 10^−4^. Homer was also used to generate RIP-seq signal plots and motif enrichment analysis.

### Splicing assays with semiquantitative RT-PCR

Total RNA was isolated and first-strand cDNA was generated as described above. PCR was performed with the KOD-Plus-Neo DNA polymerase (Toyobo). Reaction products were analyzed on 1 to 2% agarose gels with ethidium bromide staining. The amount of each splicing isoform was quantified using ImageJ software. The primers used for splicing assays are shown in table S3.

### ChIRP assay

A total of 6 × 10^7^ cells was cross-linked by 1% glutaraldehyde at room temperature for 10 min, followed by three washes in cold PBS. After being snap-frozen by liquid nitrogen and stored at −80°C, cross-linked cells were resuspended in nuclear lysis buffer [10 mM EDTA, 1% SDS, and 50 mM tris-HCl (pH 7.5)] supplemented with a PIC and sonicated until DNA was in the size range of 100 to 500 bp. Cell lysates and a set of biotin-labeled ASOs (20 nt in length) were then incubated at 37°C for 4 hours. Streptavidin-coupled Dynabeads (Invitrogen) were added to pull down the ASOs. After washing, the retrieved DNA was isolated using the chromatin immunoprecipitation DNA Clean & Concentrator kit (Zymo Research) and subjected to qPCR analysis. The ASOs used for ChIRP, as well as the primers used for qPCR detecting specific gene loci, are shown in table S3.

### RNA pull-down with biotinylated ASOs

RNA pull-down with biotin-labeled ASOs, which were the same as that used in ChIRP assay, was performed as previously descried with some modifications (*44*). Briefly, a total of 6 × 10^7^ cells were cross-linked with 254-nm UV light at a 200-mJ strength. The cells were pelleted and resuspended in radioimmunoprecipitation assay (RIPA) buffer [50 mM tris-HCl (pH 8.0), 150 mM NaCl, 5 mM EDTA, 1% NP-40, 0.1% SDS, and 1 mM DTT, supplemented with RNase OUT (50 U/ml) and a PIC] on ice for 10 min, followed by sonication for 10 min. Cell lysates was cleared by 20 min of centrifugation at 12,000*g*, and then the biotin-labeled ASOs were added and incubated at 4°C for 2 hours. Streptavidin-coupled Dynabeads (Invitrogen) were added to pull down the ASOs. After washing, the retrieved RNAs were extracted using TRIzol reagent (Invitrogen), ethanol-precipitated with GlycoBlue (Invitrogen) as a carrier, and then detected by RT-qPCR. The primers used for RT-qPCR detecting specific RNA transcripts are shown in table S3.

### 2C experiment

2C was performed as previously described with some modifications (*28*). Briefly, a total of 1 × 10^7^ cells were cross-linked with 254-nm UV light at a 150-mJ strength. Equivalent samples of nonirradiated cells were used in parallel as a negative control. The cells were homogenized in RIPA buffer [50 mM tris-HCl (pH 8.0), 150 mM NaCl, 5 mM EDTA, 1% NP-40, 0.1% SDS, and 1 mM DTT, supplemented with RNase OUT (50 U/ml) and a PIC] on ice for 10 min. Silica matrix-based columns (Foregene) were used to purify RNA and the RNA-associated proteins. After an RNase T1 treatment, the purified samples were boiled in SDS-loading buffer, and the RNA-associated proteins were separated by SDS-PAGE and detected by immunoblotting using specific Abs.

### Cell proliferation assay

A total of 3000 cells were seeded into 96-well plates and allowed to adhere to the wells. At each time point, the culture medium was replaced with CCK-8 solution (Beyotime), and then the cells were incubated at 37°C for 2 hours. The absorbance values were read at 450 nm using a Thermo Fisher Scientific Varioskan Flash multimode reader, and the cell proliferation curves were plotted using the absorbance at each time point.

### Animal studies

All experiments were conducted under protocols approved by the Institutional Animal Care and Use Committee at Sichuan University. Six-week-old female athymic BALB/c nude mice purchased from the Laboratory Animal Academy of the Sichuan Medical Sciences Institute were maintained under standardized pathogen-free conditions at the animal care facility at State Key Laboratory of Biotherapy of Sichuan University. HCC cells with indicated treatment were detached from the culture plates by a brief incubation with 2 mM EDTA in PBS, suspended in DMEM with 10% FBS, washed, and resuspended in PBS. The mice were anesthetized and subcutaneously injected into bilateral sites on the neck with 5 × 10^6^ cells in 0.1 ml of PBS using a 25-gauge needle. Tumor size was monitored every 2 days, and tumor volume was calculated by the formula *a* × *b*^2^/2 (where *a* is the longest diameter and *b* is the shortest diameter). The mice were euthanized 25 days after injection, and the tumor were excised and weighted.

### Transwell migration and invasion assay

For the migration assays, 24-well, 8-μm-diameter micropore membranes without Matrigel transwell plates (Millipore) was used. Invasion assays were conducted using 8-μm-pore, 24-well transwell plates with Matrigel (Corning). Cells were resuspended using serum-free medium. A total of 2 × 10^5^ resuspended cells were seeded into upper compartment of migration or invasion chambers, with the bottom chamber was filled with 600 μl of culture medium with 20% FBS as an attractant. After incubation in 5% CO_2_ at 37°C for 16 to 48 hours, cells that had migrated or invaded through the membrane were fixed with 75% ethanol, stained with a crystal violet, and counted under a microscope to determine cell numbers.

### Statistical analysis

Statistical analyses of experimental data were performed using the GraphPad Prism software, and those of data from TCGA and GEO repositories were implemented using R software. Student’s *t* test, log-rank test, Wilcoxon matched-paired signed rank test, Mann-Whitney *U* test, and one-way analysis of variance (ANOVA) were performed as indicated to compare the differences between experimental groups relative to their paired controls. The data were presented as the means ± SD, and *P* < 0.05 or below was considered statistically significant.

## Notes

View/request a protocol for this paper from *Bio-protocol*.
