# Supplementary material for: MALAT1 modulates alternative splicing by cooperating with the splicing factors PTBP1 and PSF
Source: Sci Adv. 2022 Dec 23;8(51):eabq7289. doi: 10.1126/sciadv.abq7289 (PMC9788761; doi:10.1126/sciadv.abq7289)
Supplement: Supplementary file 1 — Figs. S1 to S9 Tables S3 [file sciadv.abq7289_sm.pdf]

Science Advances
AAAS

Supplementary Materials for

MALAT1 modulates alternative splicing by cooperating with the splicing factors PTBP1 and PSF

Hui Miao *et al.*

Corresponding author: Ling Li, lingli1980@scu.edu.cn; Xu Song, xusong@scu.edu.cn

Sci. Adv. **8**, eabq7289 (2022)
DOI: 10.1126/sciadv.abq7289

This PDF file includes:

Figs. S1 to S9
Tables S3
Legends for tables S1 and S2

Other Supplementary Material for this manuscript includes the following:

Tables S1 and S2

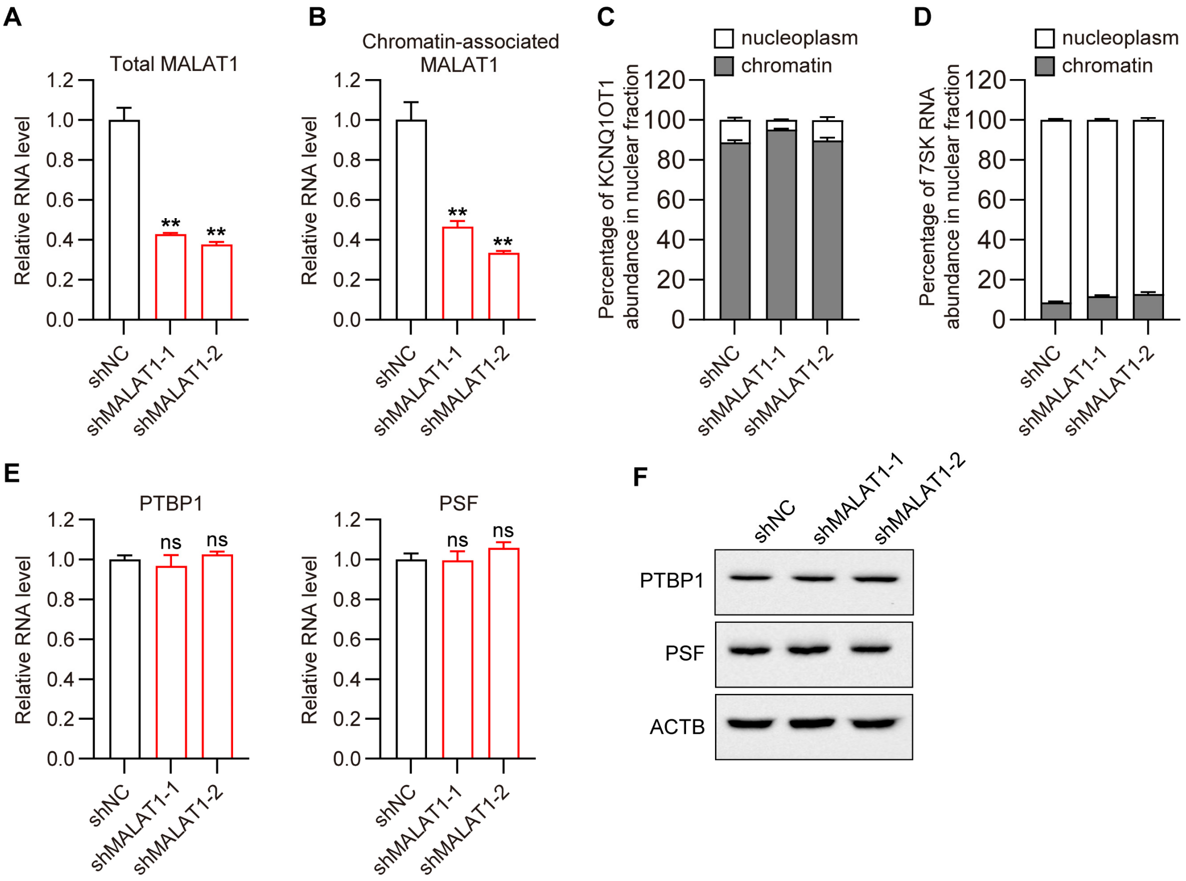

Fig. S1. Knockdown of *MALAT1* by shRNAs

(A to **D)** RT-qPCR confirming the downregulation of total (A) and chromatin-associated (B) MALAT1 by two different shRNAs in HEK293 cells. ACTB mRNA and KCNQ1OT1 RNA was included as an internal control, respectively, to normalize the amount of RNA in the samples. Distribution of KCNQ1OT1 (C) and 7SK RNA (D) was tested to confirm the separation of chromatin fraction. Data are shown as mean ± SD of n=3 independent experiments. ** $P < 0.01$ by Student's *t*-test.

(E and **F)** RT-qPCR (E) and immunoblotting (F) showing that *MALAT1* knockdown did not changes the PTBP1 and PSF levels in HEK293 cells. Data in (E) are shown as mean ± SD of n=3 independent experiments. ns, not significant.

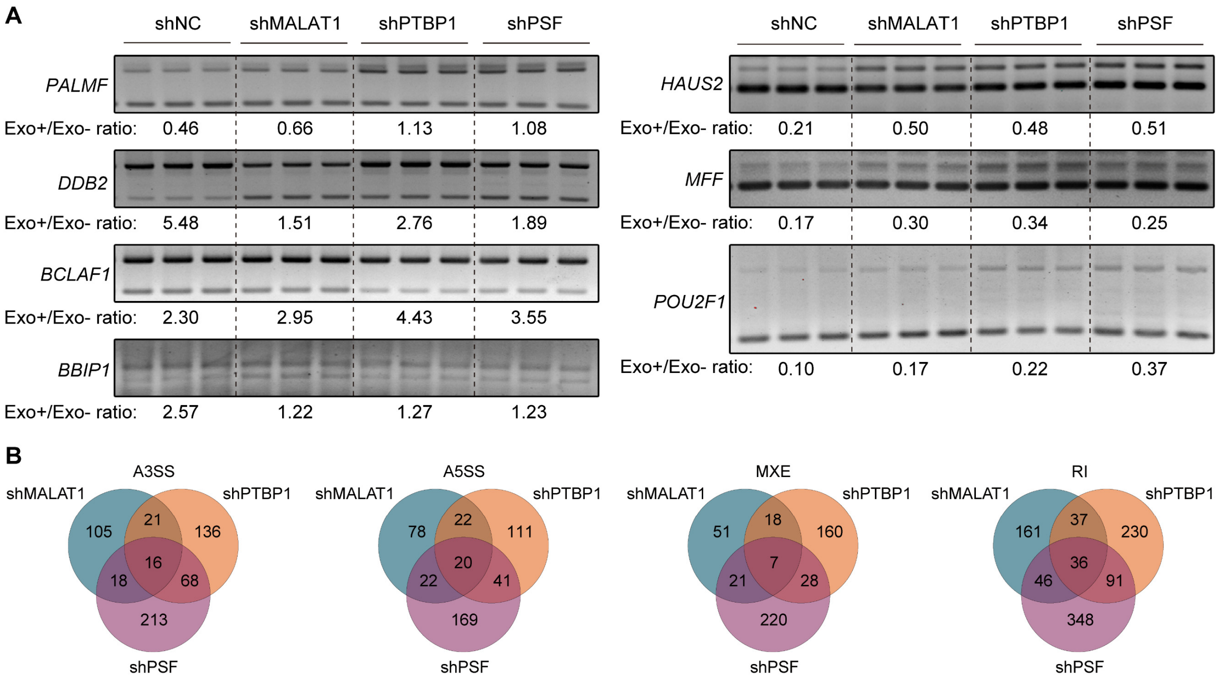

Fig. S2. Cooperative AS regulation by MALAT1, PTBP1 and PSF

(A) RT-PCR validating cassette exons co-regulated by MALAT1, PTBP1 and PSF in HEK293 cells.

(B) Venn diagrams showing overlap of different categories of AS events regulated by MALAT1, PTBP1 and PSF in HEK293 cells.

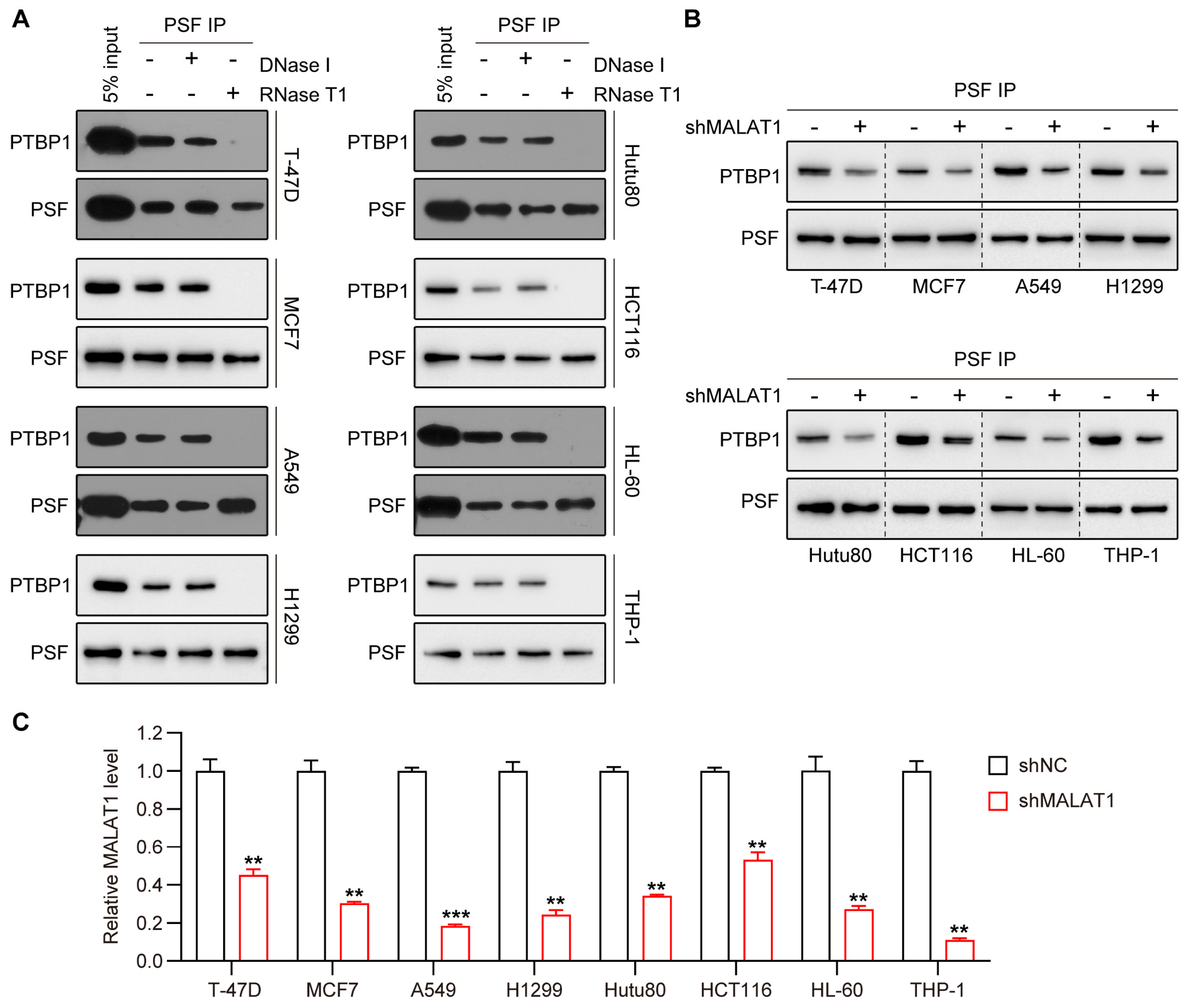

Fig. S3. MALAT1 stabilizes PTBP1/PSF interaction in multiple cellular contexts

(A) co-IP assay with anti-PSF antibody detecting the PTBP1/PSF interaction within cell extracts of the indicated tumor cells and the PTBP1/PSF interaction after DNase I or RNase T1 treatment. The dose of DNase I and RNase T1 was 15 U/reaction.

(B) co-IP assay with anti-PSF antibody detecting the PTBP1/PSF interaction in the indicated tumor cells and the PTBP1/PSF interaction after *MALAT1* knockdown.

(C) RT-qPCR validating *MALAT1* knockdown in the cells used for co-IP in (B). Data are shown as mean ± SD of n=3 independent experiments. ** $P < 0.01$, *** $P < 0.001$ by Student's *t*-test.

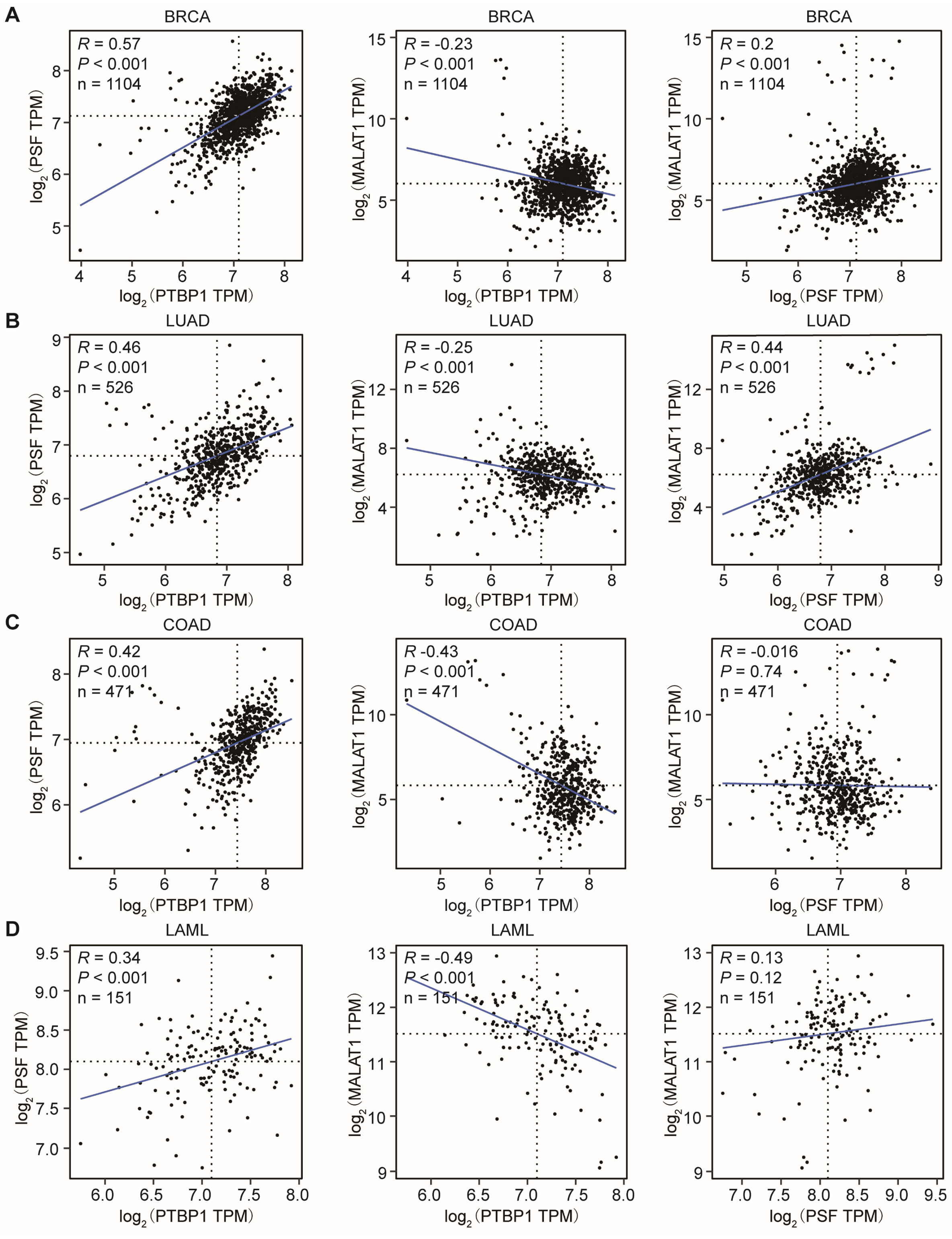
A
B
C
D
BRCA
LUAD
COAD
LAML
R = 0.57
P < 0.001
n = 1104
R = -0.23
P < 0.001
n = 1104
R = 0.2
P < 0.001
n = 1104
R = 0.46
P < 0.001
n = 526
R = -0.25
P < 0.001
n = 526
R = 0.44
P < 0.001
n = 526
R = 0.42
P < 0.001
n = 471
R -0.43
P < 0.001
n = 471
R = -0.016
P = 0.74
n = 471
R = 0.34
P < 0.001
n = 151
R = -0.49
P < 0.001
n = 151
R = 0.13
P = 0.12
n = 151
log2(PSF TPM)
log2(MALAT1 TPM)
log2(PTBP1 TPM)

Fig. S4. Correlation analysis of MALAT1, PTBP1 and PSF in different human cancers

Correlation plots showing expression levels between MALAT1, PTBP1 and PSF in BRCA (A), LUAD (B), COAD (C) and LAML (D) from the TCGA data repository.

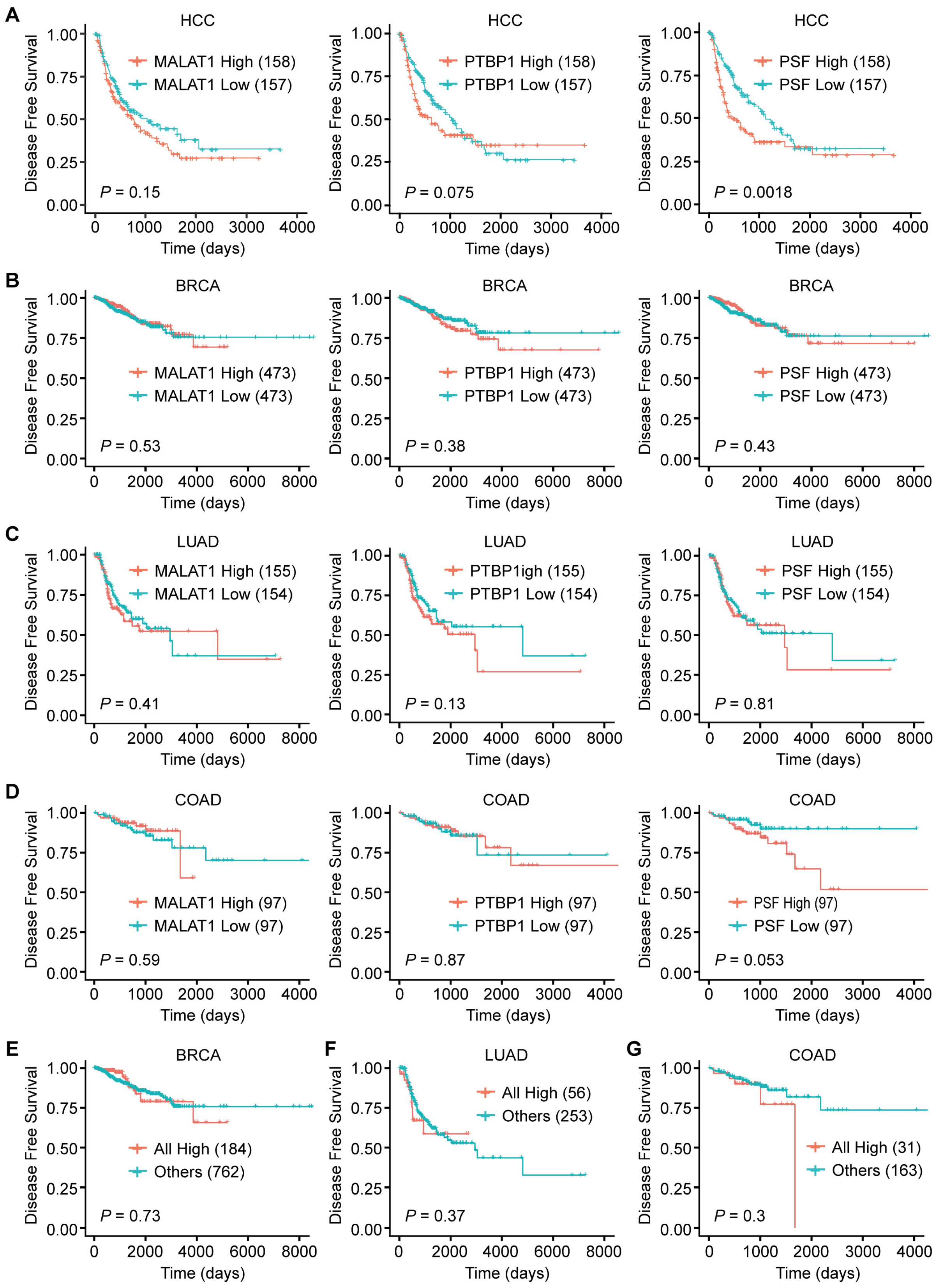

A
HCC
MALAT1 High (158)
MALAT1 Low (157)
P = 0.15
PTBP1 High (158)
PTBP1 Low (157)
P = 0.075
PSF High (158)
PSF Low (157)
P = 0.0018
B
BRCA
MALAT1 High (473)
MALAT1 Low (473)
P = 0.53
PTBP1 High (473)
PTBP1 Low (473)
P = 0.38
PSF High (473)
PSF Low (473)
P = 0.43
C
LUAD
MALAT1 High (155)
MALAT1 Low (154)
P = 0.41
PTBP1igh (155)
PTBP1 Low (154)
P = 0.13
PSF High (155)
PSF Low (154)
P = 0.81
D
COAD
MALAT1 High (97)
MALAT1 Low (97)
P = 0.59
PTBP1 High (97)
PTBP1 Low (97)
P = 0.87
PSF High (97)
PSF Low (97)
P = 0.053
E
BRCA
All High (184)
Others (762)
P = 0.73
F
LUAD
All High (56)
Others (253)
P = 0.37
G
COAD
All High (31)
Others (163)
P = 0.3
Disease Free Survival
Time (days)

Fig. S5. Prognostic values of *MALAT1*, *PTBP1* and *PSF* in different human cancers

(A to **D)** Kaplan-Meier analyses of the correlations between MALAT1, PTPB1 or PSF expression level and disease-free survival of HCC (A), BRCA (B), LUAD (C) and COAD (D) patients from TCGA panels. The median expression level was used as the cutoff. The *P*-values were determined by log-rank test.

(**E** to **G**) Kaplan-Meier analyses of the disease-free survival between BRCA (E), LUAD (F) and COAD (G) patients with high MALAT1, PTBP1 and PSF expression levels and others from TCGA panels. The median expression level was used as the cutoff. The *P*-values were determined by log-rank test.

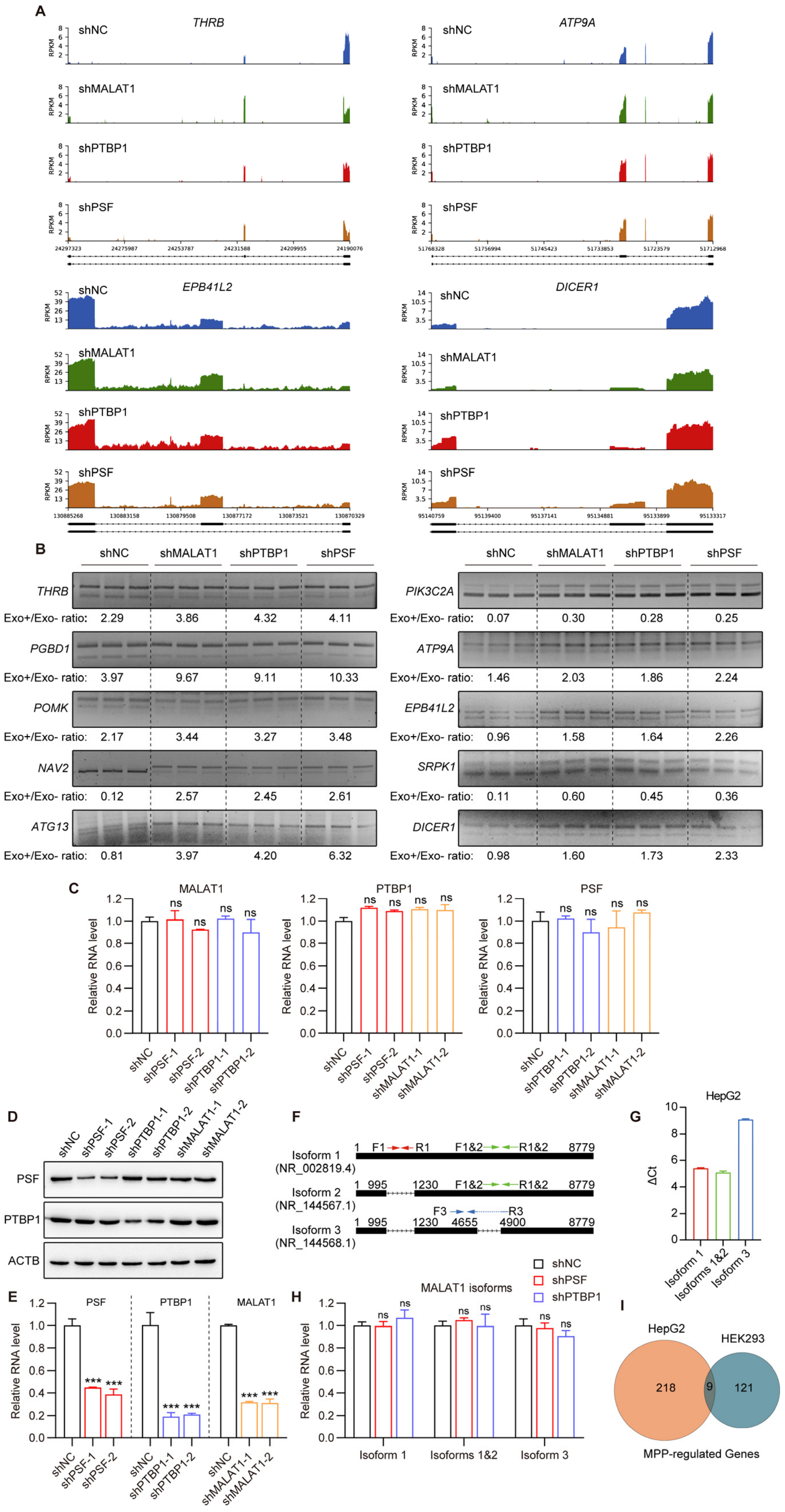
A
THRB
ATP9A
EPB41L2
DICER1
shNC
shMALAT1
shPTBP1
shPSF
RPKM
B
shNC
shMALAT1
shPTBP1
shPSF
THRB
Exo+/Exo- ratio: 2.29 3.86 4.32 4.11
PGBD1
Exo+/Exo- ratio: 3.97 9.67 9.11 10.33
POMK
Exo+/Exo- ratio: 2.17 3.44 3.27 3.48
NAV2
Exo+/Exo- ratio: 0.12 2.57 2.45 2.61
ATG13
Exo+/Exo- ratio: 0.81 3.97 4.20 6.32
PIK3C2A
Exo+/Exo- ratio: 0.07 0.30 0.28 0.25
ATP9A
Exo+/Exo- ratio: 1.46 2.03 1.86 2.24
EPB41L2
Exo+/Exo- ratio: 0.96 1.58 1.64 2.26
SRPK1
Exo+/Exo- ratio: 0.11 0.60 0.45 0.36
DICER1
Exo+/Exo- ratio: 0.98 1.60 1.73 2.33
C
MALAT1
PTBP1
PSF
Relative RNA level
ns
shNC
shPSF-1
shPSF-2
shPTBP1-1
shPTBP1-2
shMALAT1-1
shMALAT1-2
D
PSF
PTBP1
ACTB
F
Isoform 1
(NR_002819.4)
Isoform 2
(NR_144567.1)
Isoform 3
(NR_144568.1)
F1 R1 F1&2 R1&2 F3 R3
1 995 1230 4655 4900 8779
G
HepG2
ΔCt
Isoform 1
Isoforms 1&2
Isoform 3
E
***
H
MALAT1 isoforms
shNC
shPSF
shPTBP1
I
HepG2
HEK293
218
9
121
MPP-regulated Genes

Fig. S6. MALAT1 cooperates with PTBP1 and PSF to modulate pre-mRNA AS in HCC cells

(A) Representative RNA-seq signal plots within the regions around the cassette exons in MALAT1-, PTBP1- and PSF-depleted HepG2 cells relative to the control cells.

(B) RT-PCR validating the MPP-regulated exons in HepG2 cells.

(C to **E)** RT-qPCR (C) and immunoblotting (D) showing that *MALAT1*, *PTBP1* or *PSF* knockdown (E) did not change the expression level of the others. Data in (C,E) are shown as mean ± SD of n=3 independent experiments. *** $P < 0.001$ by Student's *t*-test; ns, not significant.

(**F**) Schematic diagram of MALAT1 isoforms. Arrows indicate the primers used for the subsequent RT-qPCR detecting the abundance of isoform 1, isoforms 1 & 2, and isoform 3.

(**G**) RT-qPCR detecting MALAT1 isoform expression levels in HepG2 cells. The results are shown as ΔCt values normalized to ACTB.

(**H**) RT-qPCR showing that *PTBP1* or *PSF* knockdown did not change expression level of the MALAT1 isoforms. Data are shown as mean ± SD of n=3 independent experiments. ns, not significant.

(I) Venn diagram showing overlapped MPP-regulated genes in HepG2 and HEK293 cells.

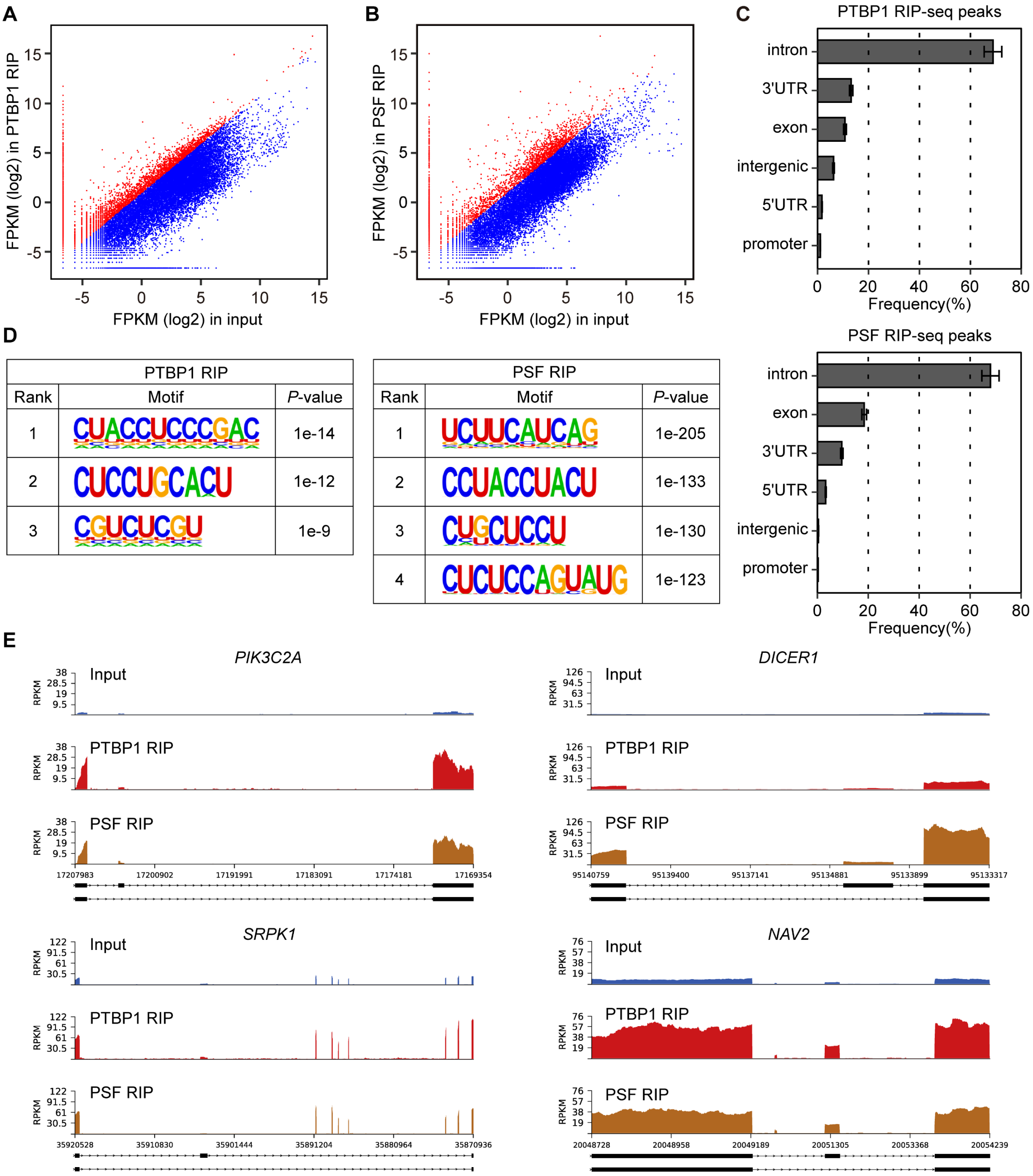

Fig. S7. MALAT1 directs association of PTBP1 and PSF with pre-mRNAs

(A and B) RIP-seq identifying transcripts that are bound by PTBP1 (A) or PSF (B) in HepG2 cells. Shown are mean adjusted FPKM + 1 (log2). Red dots show the transcripts that are enriched by

PTBP1 or PSF RIP more than 1.5-fold, and the rest transcripts are shown as blue dots. Each protein was subjected to two independent RIP-seq experiments.

(C) Position distribution of the PTBP1 and PSF RIP-seq peaks.

(D) Significantly enriched RNA motifs among the PTBP1 and PSF RIP-seq peaks.

(E) Representative RIP-seq signal plots for PTBP1 and PSF within the regions around the MPP-regulated cassette exons.

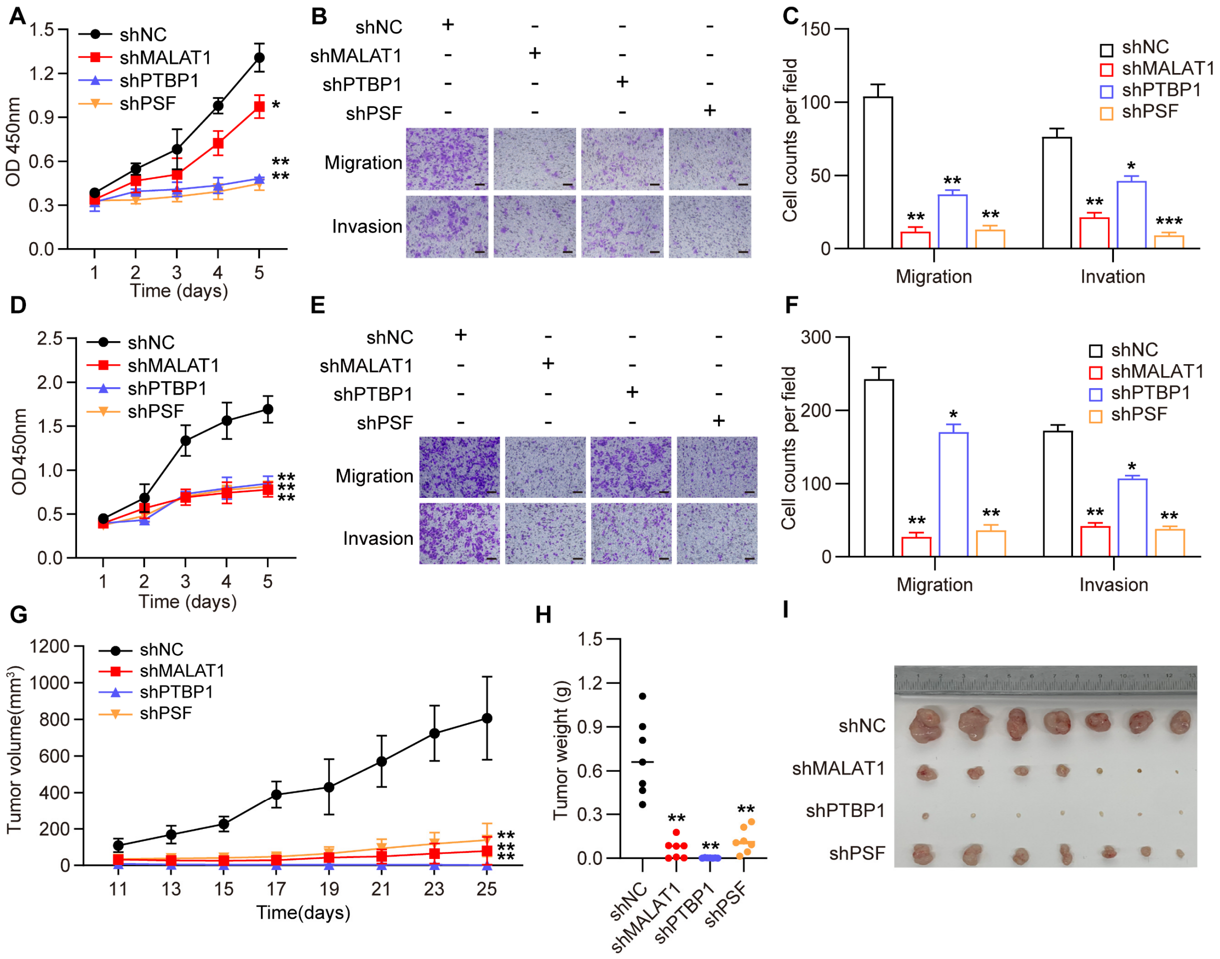

Fig. S8. MALAT1, PTBP1 and PSF contribute to the malignant property of HCC cells

(A) CCK-8 assays measuring proliferation of HepG2 cells stably depleting MALAT1, PTBP1 and PSF or control cells. Data are shown as mean ± SD of n=3 independent experiments. * $P < 0.05$, ** $P < 0.01$ by Student's *t*-test.

(B and C) Transwell migration (B, upper, C) and invasion (B, lower, C) assays of HepG2 cells stably depleting MALAT1, PTBP1 and PSF or control cells. Scale bars, 100 μm. Data in (C) are shown as mean ± SD of n=3 independent experiments. * $P < 0.05$, ** $P < 0.01$, *** $P < 0.001$ by Student's *t*-test.

(D) CCK-8 assays measuring proliferation of HCCLM3 cells stably depleting MALAT1, PTBP1 and PSF or control cells. Data are shown as mean ± SD of n=3 independent experiments. ** $P <$ 0.01 by Student's *t*-test.

(E and **F)** Transwell migration (E, upper, F) and invasion (E, lower, F) assays of HCCLM3 cells stably depleting MALAT1, PTBP1 and PSF or control cells. Scale bars, 100 μm. Data in (F) are shown as mean ± SD of n=3 independent experiments. * $P < 0.05$, ** $P < 0.01$ by Student's *t*-test.

(G to **I)** Tumor growth in subcutaneous xenografts model with HepG2 cells stably depleting MALAT1, PTBP1 and PSF or control cells. Tumor volumes were measured every 2 days (G). The mice were killed 25 days after injection, and the tumor were excised and weighted (H,I). Data in (G,H) are shown as mean ± SD of n=7 mice in each group. ** $P < 0.01$ by Mann-Whitney *U*-test.

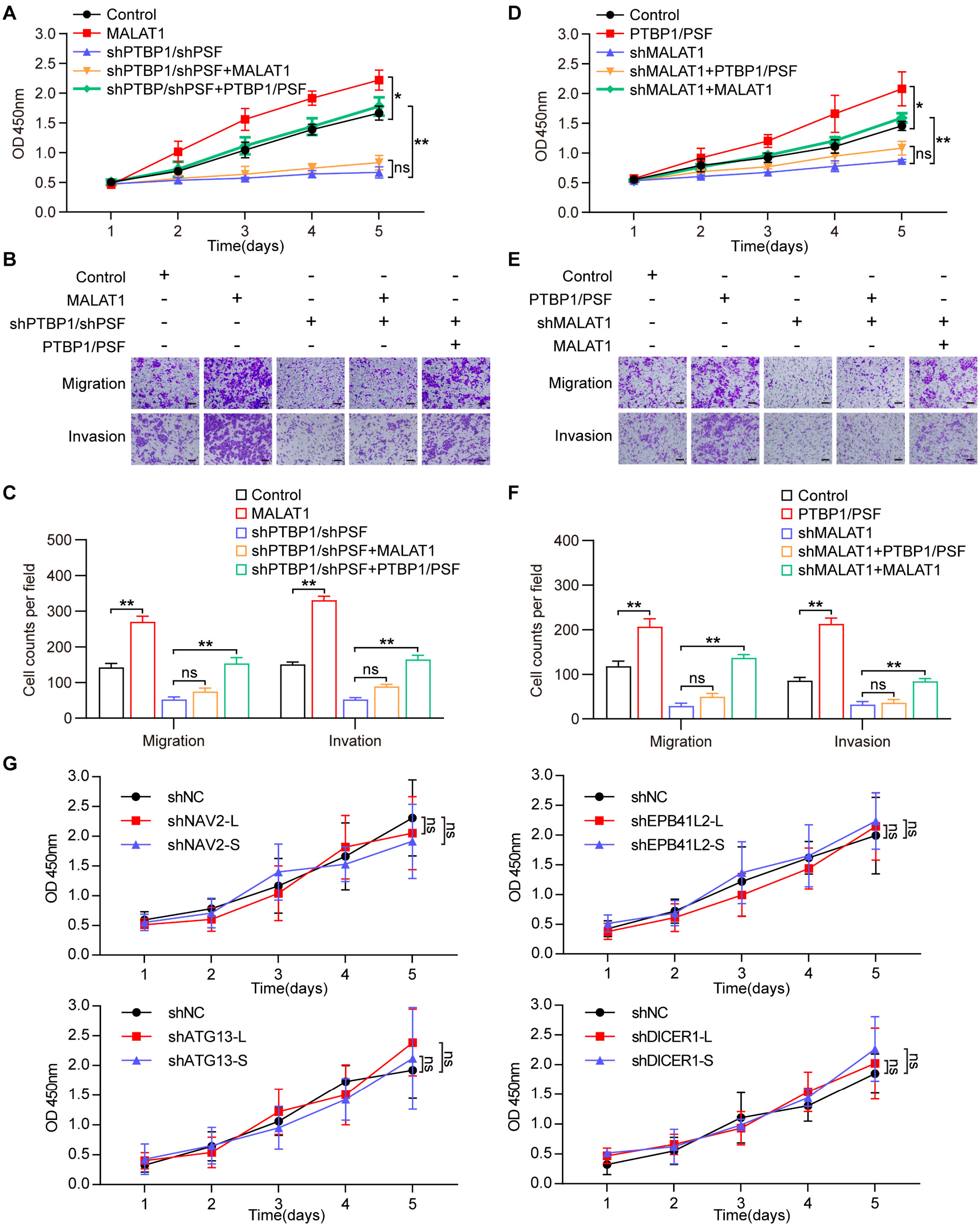

A
Control
MALAT1
shPTBP1/shPSF
shPTBP1/shPSF+MALAT1
shPTBP/shPSF+PTBP1/PSF
OD 450nm
Time(days)
ns
D
Control
PTBP1/PSF
shMALAT1
shMALAT1+PTBP1/PSF
shMALAT1+MALAT1
OD 450nm
Time(days)
ns
B
Control
MALAT1
shPTBP1/shPSF
PTBP1/PSF
Migration
Invasion
E
Control
PTBP1/PSF
shMALAT1
MALAT1
Migration
Invasion
C
Control
MALAT1
shPTBP1/shPSF
shPTBP1/shPSF+MALAT1
shPTBP1/shPSF+PTBP1/PSF
Cell counts per field
ns
Migration
Invation
F
Control
PTBP1/PSF
shMALAT1
shMALAT1+PTBP1/PSF
shMALAT1+MALAT1
Cell counts per field
ns
Migration
Invasion
G
shNC
shNAV2-L
shNAV2-S
OD 450nm
Time(days)
shNC
shEPB41L2-L
shEPB41L2-S
OD 450nm
Time(days)
shNC
shATG13-L
shATG13-S
OD 450nm
Time(days)
shNC
shDICER1-L
shDICER1-S
OD 450nm
Time(days)
ns

Fig. S9. MALAT1, PTBP1 and PSF have synergistic effect on malignant behavior of HCC cells

(A) CCK-8 assays detecting implication of PTBP1 and PSF in the regulatory effect of MALAT1 on proliferation of HCCLM3 cells. Data are shown as mean ± SD of n=3 independent experiments. * $P < 0.05$, ** $P < 0.01$ by one-way ANOVA; ns, not significant.

(B and **C)** Transwell assays detecting implication of PTBP1 and PSF in the regulatory effect of MALAT1 on migration (B, upper, C) and invasion (B, lower, C) of HCCLM3 cells. Scale bars, 100 μm. Data in (C) are shown as mean ± SD of n=3 independent experiments. ** $P < 0.01$ by one-way ANOVA; ns, not significant.

(D) CCK-8 assays detecting contribution of MALAT1 to the regulatory effect of PTBP1 and PSF on proliferation of HCCLM3 cells. Data are shown as mean ± SD of n=3 independent experiments. * $P < 0.05$, ** $P < 0.01$ by one-way ANOVA; ns, not significant.

(E and **F)** Transwell assays detecting contribution of MALAT1 to the regulatory effect of PTBP1 and PSF on migration (E, upper, F) and invasion (E, lower, F) of HCCLM3 cells. Scale bars, 100 μm. Data in (F) are shown as mean ± SD of n=3 independent experiments. ** $P < 0.01$ by one-way ANOVA; ns, not significant.

(**G**) CCK-8 assays measuring proliferation of HepG2 cells depleting the transcript variants of *NAV2*, *EPB41L2*, *ATG13* and *DICER1* or control cells. Data are shown as mean ± SD of n=3 independent experiments. ns, not significant.

Table S1. List of PTBP1-binding sites detected by RIP-seq

Table S2. List of PSF-binding sites detected by RIP-seq

Table S3. Sequence of primers and oligos used in this study

Name (F: forward, R: reverse)	Sequences (5′ to 3′)
**RT-qPCR for gene expression assay**	
7SK RNA-F	ATCGCCAGGGTTGATTCGGCTGAT
7SK RNA-R	GGATGTGTCTGGAGTCTTGGAAGC
ACTB-F	GCACTCTTCCAGCCTTCCTTC
ACTB-R	TTGGCGTACAGGTCTTTGCGGA
PTBP1-F	CTTCTACCCTGTGACCCTGGATGTG
PTBP1-R	CTGGTTGTTCTTGGTGAAGGTGATG
PSF-F	CGCCAAGATCTGATGAGACGACAG
PSF-R	GCCTCATTTGTTCTTCCATCTCACG
MALAT1-F	GGATCCTAGACCAGCATGCC
MALAT1-R	AAAGGTTACCATAAGTAAGTTCCAGAAAA
MALAT1-isoform1-F	GGGTTTTGTGAGGTGTTTGATGACC
MALAT1-isoform1-R	TAGCGTGTGGAAAGATTTGAGCTGC
MALAT1-isoform1&2-F	GCTTGAATGTCTCTTAGAGGGTGGG
MALAT1-isoform1&2-R	TACTGCCAGGCTGGTTATGACTCAG
MALAT1-isoform3-F	TAGAAACTGCAGAGCAAAGGAAGTG
MALAT1-isoform3-R	CTCAGAAGATGTTATCTGAAAAAAGTTTG
NEAT1-F	ATGCCACAACGCAGATTGAT
NEAT1-R	CGAGAAACGCACAAGAAGG
**RT-PCR for splicing assay**	
PALMF-F	ACAAGCGAGTCTCCAACAC
PALMF-R	GGATGAGTTCGTCCACCTC
DDB2-F	GCCTGGATGTGTCTGCTAGT
DDB2-R	TGCTGGAAGTGACGGTGAG
BCLAF1-F	ATCCTCTTCAGCATCACCTTCT
BCLAF1-R	CCAATAATCCACACCATCATCTCT
BBIP1-F	GGTAATACCGGGTTGGATGCT
BBIP1-R	CTTCCCGGAACATTGACTTCAC
HAUS2-F	ACCTGGAAATTGAACTCCTGAAACTAG
HAUS2-R	GTCTTTGCCTAAGGGATCTCTTCTCT
MFF-F	CGAGCAGTTGGCAGACTAAA
MFF-R	ATGAGGATTAGAAGTGGCGG
POU2F1-F	CCGTCAGAAACCAGTAAACCAT
POU2F1-R	GTTGAGATTGCTCCTCCTACAG
THRB-F	AGAGGTTAGCAGTAGACTGTTGGAAG
THRB-R	TGTGGCGACGACTGTTCATTT

PGBD-F	ACTCCGAGAACTTTGTCATC
PGBD-R	TGGGTTAAACACAGGCACTA
POMK-F	CAGGAGCCGCAGAGGCTTGG
POMK-R	CGAGGAGCGATGAAGAAGTGGT
NAV2-F	GGGAAGCCTACCAAGCAAGTG
NAV2-R	GCAGAATGGGACCGTAACCA
ATG13-F	AAGCCTTAGGTGTCTATCGG
ATG13-R	ATCATGTCATTGCACTCCAG
PIK3C2A-F	AAATGGCTCGGACTGTGGCG
PIK3C2A-R	CCTCTGCTTCCATCTGTAATGCTTCT
ATP9A-F	GGATGGACAGCAGGCCCCGC
ATP9A-R	GGCTGTAGACCTGGGAGTTG
EPB41L2-F	TGGGGAGATTTCAGCCTATGG
EPB41L2-R	CTTCTGACTCGGGAGCACGT
SRPK1-F	CTCCAGGCCCGAAAGAAAAG
SRPK1-R	GGTTTAGGCTGGGGAGCAGTA
DICER1-F	ATGCAGTTCAGACAAGAGCAACAC
DICER1-R	GCAGGGCTTTTCATTCATCCA
**RT-qPCR for splicing assay**	
DICER1-L-F	GCTGACAGTAAAGCCAGTGGTAGG
DICER1-L-R	GAAAGGACCCATTGGTGAGGAA
DICER1-S-F	CTCAAACATTAAAACGTAAGCTGTGCT
DICER1-S-R	AGGACCCATTGGTGAGGAAGC
ATG13-L-F	TCTGAAGCCTTAGGTGTCTATCGG
ATG13-L-R	CACAGGTCGTTCCACTGCGA
ATG13-S-F	TCTGAAGCCTTAGGTGTCTATCGG
ATG13-S-R	GTGACAAAGCAAAACCCTGCGA
NAV2-L-F	ACCACACTGTCAGAAAGTGA
NAV2-L-R	GGAATTGGCAGCGGTGTCCT
NAV2-S-F	GTCCCCAGCCACAATTCTTCC
NAV2-S-R	GTGGGAGTATACCTTTCTGA
EPB41L2-L-F	GAACTGGATAAGGCCCAGGAGG
EPB41L2-L-R	CCATAAAATTGCGCTTGAGTTCACTA
EPB41L2-S-F	TGGGGAGATTTCAGCCTATGGA
EPB41L2-S-R	TCGGGAGCACGTGTCTTTCCT
**RT-qPCR for pre-mRNA detection**	
THRB-F	TGATGATGTGAACGACCAGAGTG
THRB-R	CAACGTGAAACCGAAACAAGC
PGBD-F	TGTGAAAGTGAAGGAGGAAGATCC
PGBD-R	GGTAGCAGAAGTGCCGAAAGC
POMK-F	GGTTCATTTGCGATTGCTGT
POMK-R	AGGGCTGAAACCTATGCCTACT
NAV2-F	TTTGGGGAGCAGGTGGTTT
NAV2-R	GACACTGAGCGGGATGTGACTA
ATG13-F	GGTTAGGTCGTCAGGCGTTTT
ATG13-R	AACAGTCACCCTCACCTACACG

PIK3C2A-F	TATTCGGGGTCCTGCTCTGCAA
PIK3C2A-R	CCTCCTTCTGTGGGACAAAACTAACT
ATP9A-F	CCTCTTGGTGTTGGTCTTGTCA
ATP9A-R	GGCAGAACGGGAAAGGGATT
EPB41L2-F	CGCCCTAATGAATGGGAAAA
EPB41L2-R	CTGAAGTGAGACAGAGCCAACAAA
SRPK1-F	AGACTTCTGCAATGCTATCAGCC
SRPK1-R	CCAGTTTAACTTAACAGCCATCACTA
DICER1-F	GTCCTATCAGATCAGGGGAGACT
DICER1-R	CCAAAAGCAATCCATTCAAAA
**Oligos for ChIRP assay**	
lacZ probes	TCACGACGTTGTAAAACGAC
	AGGTTACGTTGGTGTAGATG
	GTAGCCAGCTTTCATCAACA
	AGATGAAACGCCGAGTTAAC
	TTTCTCCGGCGCGTAAAAAT
	AACGAGACGTCACGGAAAAT
	TTAAAGCGAGTGGCAACATG
	ATAATTTCACCGCCGAAAGG
	ATAGAGATTCGGGATTTCGG
	TTAACGCCTCGAATCAGCAA
	CACGGCGTTAAAGTTGTTCT
	TTGGCTTCATCCACCACATA
	GATCACACTCGGGTGATTA
	AAATAATATCGGTGGCCGTG
	ATTTAGCGAAACCGCCAAGA
	TTAATCAGCGACTGATCCAC
	AAACTGCTGCTGGTGTTTTG
	GTTATCGCTATGACGGAACA
	GTTCAGGCAGTTCAATCAAC
	TTGCCAACGCTTATTACCCA
MALAT1 probes	AGATATTGTGCTGTTACCTC
	CCTACTGAAGAGCATTGGAG
	ATAGAGCTACTTAGCTGTGG
	AAGCTAGGGAAAAGTGGTTG
	AAATCAGGTGAGGCTGACAC
	AACACAGTTTGCTCACATGC
	TTCTCTGGCCCTTCGCATAC
	TCCCACCACCAGAAATGAAC
	GTCGTTTCACAATGCATTCT
	CACTGCAAGGTCTCATACAC
	AAACATTGCCTACCACTCTA
	TATTCCTTATTTAGAGGGCC
	CCCAAGATTGCCCCAACACT
	TCTGATTCTAACAGCACATC

	AGTGTACTATCCCATCACTG<br>ATACCTGTCTGAGGCAAACG<br>GTGGTTATAGCTTGACAAGC<br>CCCAATGGAGGTATGACATA<br>CACCCTCTAAGAGACATTCA<br>CTTCAGGATCATTAAGCCAC<br>ATCATTGGGAGTTACTTGCC<br>GGCCTACTCAAGCTCTTCTG<br>CTCTAACCCAGTTTGTCAAT<br>AACTGTAAACCTGTGGTGGT<br>CCTCAGTTACACATCCAAAC<br>TTGTCTCAATTTGGCTATCA<br>TCCCTTTACACCTCAGTACG<br>CTTCACCACCAAATCGTTAG<br>TGTGTAGCACCTGGGTCAGC<br>TCTAACTTCTGCACCACCAG<br>GTACATTTTGCCCTTAGCTT<br>CGTCATGGATTTCAAGGTCT<br>TCATCTCAACCTCCGTCATG<br>GCGAGGCGTATTTATAGACG<br>AACGGGTCATCAAACACCTC<br>GCTTGCAGTCCTGCGACTTG<br>CCAGTGGCCCACTCTGATCT<br>CATGGAAAGCGAGTTCAAGT<br>TATGAGCTTCAGACCTTCTG<br>TGGGCTCCCGGAGGCGTCAG
**Oligos encoding shRNAs**	
shNC sense	CCGGGCGCGATAGCGCTAATAATTTCTCGAGAAATTAT<br>TAGCGCTATCGCGCTTTTTG
shNC antisense	AATTCAAAAAGCGCGATAGCGCTAATAATTTCTCGAGA<br>AATTATTAGCGCTATCGCGC
shMALAT1-1 sense	CCGGGCTGTGGAGTTCTTAAATATCCTCGAGGATATTT<br>AAGAACTCCACAGCTTTTTG
shMALAT1-1 antisense	AATTCAAAAAGCTGTGGAGTTCTTAAATATCCTCGAGG<br>ATATTTAAGAACTCCACAGC
shMALAT1-2 sense	CCGGGAGCAAAGGAAGTGGCTTAATCTCGAGATTAAG<br>CCACTTCCTTTGCTCTTTTTG
shMALAT1-2 antisense	AATTCAAAAAGAGCAAAGGAAGTGGCTTAATCTCGAG<br>ATTAAGCCACTTCCTTTGCTC
shPSF-1 sense	CCGGAAGGCAAAGGATTCGGATTTACTCGAGTAAATCC<br>GAATCCTTTGCCTTTTTTTG
shPSF-1 antisense	AATTCAAAAAAAGGCAAAGGATTCGGATTTACTCGAGT<br>AAATCCGAATCCTTTGCCTT

shPSF-2 sense	CCGGGGAAGATGCCTATCATGAACACTCGAGTGTTCATGATAGGCATCTTCCTTTTTG
shPSF-2 antisense	AATTCAAAAAGGAAGATGCCTATCATGAACACTCGAGTGTTCATGATAGGCATCTTCC
shPTBP1-1 sense	CCGGAACTTCCATCATTCCAGAGAACTCGAGTTCTCTGGAATGATGGAAGTTTTTTTG
shPTBP1-1 antisense	AATTCAAAAAAACTTCCATCATTCCAGAGAACTCGAGTTCTCTGGAATGATGGAAGTT
shPTBP1-2 sense	CCGGCAAGAACTTCCAGAACATATTCTCGAGAATATGTTCTGGAAGTTCTTGTTTTTG
shPTBP1-2 antisense	AATTCAAAAACAAGAACTTCCAGAACATATTCTCGAGAATATGTTCTGGAAGTTCTTG
sh7SK sense	CCGGGCTCTCAAGGTCCATTTGTAGCTCGAGCTACAAATGGACCTTGAGAGCTTTTTG
sh7SK antisense	AATTCAAAAAGCTCTCAAGGTCCATTTGTAGCTCGAGCTACAAATGGACCTTGAGAGC
shNEAT1 sense	CCGGCATGGACCGTGGTTTGTTACTCTCGAGAGTAACAAACCACGGTCCATGTTTTTG
shNEAT1 antisense	AATTCAAAAACATGGACCGTGGTTTGTTACTCTCGAGAGTAACAAACCACGGTCCATG
shNAV2-L sense	CCGGAAGAAAGGACTCAGGTATACTCTCGAGAGTATACCTGAGTCCTTTCTTTTTTTG
shNAV2-L antisense	AATTCAAAAAAAGAAAGGACTCAGGTATACTCTCGAGAGTATACCTGAGTCCTTTCTT
shNAV2-S sense	CCGGACACTGTCAGAAAGGTATACTCTCGAGAGTATACCTTTCTGACAGTGTTTTTTG
shNAV2-S antisense	AATTCAAAAAACACTGTCAGAAAGGTATACTCTCGAGAGTATACCTTTCTGACAGTGT
shEPB41L2-L sense	CCGGGGAGGACATACTGAAACATCACTCGAGTGATGTTTCAGTATGTCCTCCTTTTTG
shEPB41L2-L antisense	AATTCAAAAAGGAGGACATACTGAAACATCACTCGAGTGATGTTTCAGTATGTCCTCC
shEPB41L2-S sense	CCGGGCTCATTGAAGGAAAGGACACCTCGAGGTGTCCTTTCCTTCAATGAGCTTTTTG
shEPB41L2-S antisense	AATTCAAAAAGCTCATTGAAGGAAAGGACACCTCGAGGTGTCCTTTCCTTCAATGAGC
shDICER1-L sense	CCGGGGTAGGCTTTCACACAGATATCTCGAGATATCTGTGTGAAAGCCTACCTTTTTG
shDICER1-L antisense	AATTCAAAAAGGTAGGCTTTCACACAGATATCTCGAGATATCTGTGTGAAAGCCTACC
shDICER1-S sense	CCGGAACGGTAAGCTGTGCTAGAACCTCGAGGTTCTAGCACAGCTTACCGTTTTTTTG
shDICER1-S antisense	AATTCAAAAAAACGGTAAGCTGTGCTAGAACCTCGAGGTTCTAGCACAGCTTACCGTT
shATG13-L sense	CCGGGCTTCGCAGGTGGAACGACCTCTCGAGAGGTCGTTCCACCTGCGAAGCTTTTTG

shATG13-L antisense	AATTCAAAAAGCTTCGCAGGTGGAACGACCTCTCGAGAGGTCGTTCCACCTGCGAAGC
shATG13-S sense	CCGGTTCGCAGGGCTGGAGTGCAATCTCGAGATTGCACTCCAGCCCTGCGAATTTTTG
shATG13-S antisense	AATTCAAAAATTCGCAGGGCTGGAGTGCAATCTCGAGATTGCACTCCAGCCCTGCGAA
